# Dissecting the antibacterial activity of oxadiazolone-core derivatives against *Mycobacterium abscessus*

**DOI:** 10.1371/journal.pone.0238178

**Published:** 2020-09-18

**Authors:** Abdeldjalil Madani, Ivy Mallick, Alexandre Guy, Céline Crauste, Thierry Durand, Patrick Fourquet, Stéphane Audebert, Luc Camoin, Stéphane Canaan, Jean François Cavalier

**Affiliations:** 1 Aix-Marseille Univ., CNRS, LISM, Institut de Microbiologie de la Méditerranée FR3479, Marseille, France; 2 IHU Méditerranée Infection, Aix-Marseille Univ., Marseille, France; 3 IBMM, Univ Montpellier, CNRS, ENSCM, Montpellier, France; 4 Aix-Marseille Univ, INSERM, CNRS, Institut Paoli-Calmettes, CRCM, Marseille Protéomique, Marseille, France; Institut de Pharmacologie et de Biologie Structurale, FRANCE

## Abstract

*Mycobacterium abscessus (M*. *abscessus)*, a rapidly growing mycobacterium, is an emergent opportunistic pathogen responsible for chronic bronchopulmonary infections in individuals with respiratory diseases such as cystic fibrosis. Most treatments of *M*. *abscessus* pulmonary infections are poorly effective due to the intrinsic resistance of this bacteria against a broad range of antibiotics including anti-tuberculosis agents. Consequently, the number of drugs that are efficient against *M*. *abscessus* remains limited. In this context, 19 oxadiazolone (**OX**) derivatives have been investigated for their antibacterial activity against both the rough (R) and smooth (S) variants of *M*. *abscessus*. Several **OXs** impair extracellular *M*. *abscessus* growth with moderated minimal inhibitory concentrations (MIC), or act intracellularly by inhibiting *M*. *abscessus* growth inside infected macrophages with MIC values similar to those of imipenem. Such promising results prompted us to identify the potential target enzymes of the sole extra and intracellular inhibitor of *M*. *abscessus* growth, *i*.*e*., compound **iB*p*PPOX**, *via* activity-based protein profiling combined with mass spectrometry. This approach led to the identification of 21 potential protein candidates being mostly involved in *M*. *abscessus* lipid metabolism and/or in cell wall biosynthesis. Among them, the Ag85C protein has been confirmed as a vulnerable target of **iB*p*PPOX**. This study clearly emphasizes the potential of the **OX** derivatives to inhibit the extracellular and/or intracellular growth of *M*. *abscessus* by targeting various enzymes potentially involved in many physiological processes of this most drug-resistant mycobacterial species.

## Introduction

Non-tuberculous mycobacteria (NTM) are naturally-occurring bacterial species mostly found in soil and water that do not cause tuberculosis or leprosy [1]. NTM are opportunistic pathogens able to infect humans with predisposing conditions like cystic fibrosis (CF) or immunosuppression and responsible for wide range of infections like skin infections, pulmonary infections or disseminated diseases [2–4]. In the last decades, NTM infections are increasing worldwide, the most frequently reported species being *Mycobacterium avium* complex (MAC) and *M*. *abscessus* complex [3, 5].

*M*. *abscessus* can be isolated from solid medium with either a smooth (S) or a rough (R) colony morphotype [6]. The difference between both morphotypes is related to the presence of glycopeptidolipids (GPLs) in the cell wall of the S variant, while absent in the R one [7]. This latter R strain is also associated with severe and persistent infections [8]. In CF patients, treatment of *M*. *abscessus* complex infections requires a multidrug therapy including a daily oral macrolide (clarithromycin or azythromicin) in conjunction with intravenous amikacin and a β-lactam (imipenem or cefoxitin) [9]. However, almost 60% of *M*. *abscessus* strains could develop both intrinsic and acquired resistance to currently available antibiotics, including macrolides [4, 10]. As a direct consequence, treatment of such infections has become very complicated with very limited alternative options [5, 11].

**Fig 1:**
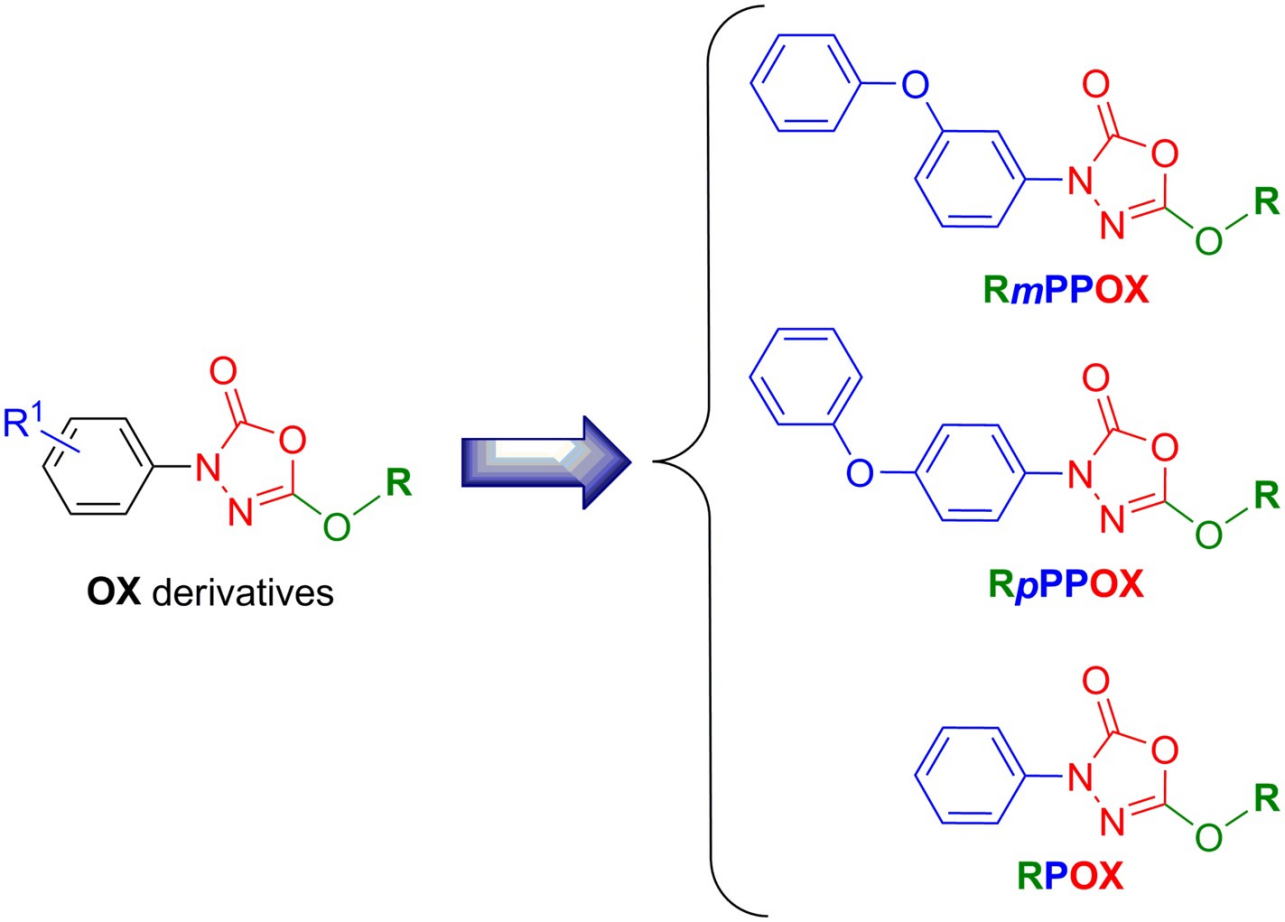
Chemical structure of the OX derivatives. **R*m***(or ***p***)**PPOX** nomenclature is as follows: ***m*** (or ***p***)**P** represents the *meta* (or *para*)-Phenoxy group when present; **P** the phenyl group; **OX** the Oxadiazolone core; and **R** the alkyl chain (*i*.*e*., **M**; methyl, **E**, ethyl; **B**, butyl; **iB**, isobutyl; **H**, hexyl; **O**, octyl; **Eh**, 2-ethylhexyl; **D**, decyl; **Do**, dodecyl; **Be**, benzyloxyethyl; **Me**, methoxyethyl). Adapted from [12].

Due to the worldwide increasing incidence and prevalence of *M*. *abscessus* and the inherent difficulties to manage such resistant pulmonary infections, new active molecules are urgently needed. In this context, we recently investigated the antibacterial activities of 19 oxadiazolone-core (**OX**) derivatives (Fig 1) against three pathogenic slow-growing mycobacteria: *M*. *marinum*, *M*. *bovis* BCG as well as *M*. *tuberculosis* H37Rv the etiologic agent of tuberculosis [12].

These **OX** compounds exhibited not only encouraging minimal inhibitory concentrations (MIC), but above all, they were also found to display a diversity of actions by acting either only on extracellular *M*. *tuberculosis* growth, or both intracellularly on infected macrophages as well as extracellularly on bacterial growth. Remarkably, all **OX** derivatives exhibited very low toxicity towards host cell macrophages [12]. Of interest, only the **iB*p*PPOX** derivative exhibited moderate (MIC_50_ = 32.0 μM) to quite good (MIC_50_ = 8.5 μM) antibacterial activity against both extracellular and intramacrophagic *M*. *tuberculosis* H37Rv, respectively [12]. Following an activity-based protein profiling (ABPP) approach combined with mass spectrometry, 18 putative target(s) of **HPOX**, a selective inhibitor of *M*. *tuberculosis* extracellular growth, were identified. All these proteins were (Ser/Cys)-enzymes possessing a catalytic serine or cysteine residue, and involved in *M*. *tuberculosis* lipid metabolism and/or in cell wall biosynthesis. Above all, the results of this study imply that such **OX** derivatives represent a novel class of multi-target mycobacterial inhibitors *via* the formation of a covalent bond with the catalytic residue of various mycobacterial (Ser/Cys)-containing enzymes involved in various physiological processes.

Given all these previous findings, in the present study we have further assessed the antibacterial activity of these 19 **OXs** against *M*. *abscessus* growth. The determined MIC revealed that some **OXs** were able to inhibit *M*. *abscessus* growth *in vitro* in culture broth medium and/or intracellularly inside macrophages. In addition, using a similar ABPP assay as previously reported for *M*. *tuberculosis* [12], the potential target enzymes of **iB*p*PPOX**, the most active inhibitor of extra- and intracellular bacterial growth, were further identified.

## Materials and methods

### Bacterial strains and growth conditions

*M*. *abscessus* CIP104536^T^ with either a smooth (S) or rough (R) morphotype was grown in Middlebrook 7H9 broth (BD Difco, Le Pont de Claix, France) supplemented with 0.2% glycerol, 0.05% Tween 80 and 0.2% glucose (Sigma-Aldrich, St. Quentin Fallavier, France) (7H9-S).

### Chemicals

Clarythromycine and Imipenem mixture w/Cilastatin were purchased from Euromedex (Souffelweyersheim, France). The Oxadiazolone derivatives were synthesized as previously reported and were at least 98% pure as determined by HPLC analysis [12]. Stock solutions of each inhibitor (4 mg/mL) were prepared in DMSO and stored at -20 °C before use.

### Resazurin microtiter assay (REMA) for MIC determination—Extracellular assay

Susceptibility testing was performed using the Middlebrook 7H9 broth microdilution method. MICs of the **OXs** were determined in 96-well flat-bottom Nunclon Delta Surface microplates with lid (Thermo-Fisher Scientific, ref. 167008) using the resazurin microtiter assay (REMA) [12–15]. Briefly, log-phase bacteria were diluted to a cell density of 5 × 10^6^ cells/mL and 100 μL of this inoculum was grown in a 96-well plate in the presence of serial dilutions of each **OX** compound. After 3–5 days incubation at 37 °C, 20 μL of a 0.025% (*w/v*) resazurin solution was added to each well (200 μL) and incubation was continued until the appearance of a color change (from blue to pink) in the control well (*i*.*e*., bacteria without antibiotics). Fluorescence of the resazurin metabolite resorufin (λ_excitation_, 530 nm; λ_emission_, 590 nm) was then measured [13, 16] and the concentration leading to 50% and 90% growth inhibition was defined as the MIC_50_ and MIC_90_, respectively. See S1 Appendix for detailed protocol.

### Intramacrophage killing assay—Intracellular assay

The intracellular growth of *M*. *abscessus* S was assessed following a 24 h exposure of infected Raw264.7 murine macrophages cell line (American Type Culture Collection TIB-71) to each of the 19 **OX** compounds at a final concentration of 30 μM [17]. To avoid growth of extracellular mycobacteria, cells were extensively washed and treated with amikacin (200 μg/mL = 340 μM; 87 × MIC_50_) prior to treatment with the **OX** analogs. Imipenem (IMP; 80 μg/mL = 267 μM; 64 × MIC_50_) was used as positive control for this intracellular killing assay. In each case, the viability of infected macrophages was checked by addition of trypan blue [18] before cell lysis and plating for CFU count. See S1 Appendix for detailed protocol.

### iB*p*PPOX target enzymes identification

#### Activity-Based Protein Profiling (ABPP) for the identification of iB*p*PPOX target enzymes

Bacterial suspension of *M*. *abscessus* R in 7H9-S was adjusted at an OD_600_ corresponding to 6×10^9^ cells/mL and then incubated with **iB*p*PPOX** inhibitor (400 μM final concentration) or DMSO (control) at 37 °C for 2–3 h. under gentle shaking at 75 rpm. Bacteria were then washed 3 times with PBS containing 0.05% Tween 80, resuspended in PBS buffer at a 1:1 (*w/v*) ratio and then lysed by mechanical disruption on a BioSpec Beadbeater. Both **iB*p*PPOX**-treated *M*. *abscessus* and DMSO-control lysate samples (750 μL– 0.75 mg total proteins) were labeled with 2 μM Desthiobiotin-FP probe for 90 min at room temperature. Samples were enriched for biotinylated proteins using 0.8 μm Nanolink streptavidin magnetic beads (Solulink), according to the manufacturer’s instructions. The resulting captured biotinylated proteins solution was mixed with 5X Laemmli reducing sample buffer, and heated at 95 °C for 5 min. The released denatured proteins were subjected to tryptic digestion, peptide extraction, and LC-MS/MS analysis as described below.

Alternatively, *M*. *abscessus* R total lysates (500 μL − 1 mg total proteins) were further pre-incubated with **iB*p*PPOX** (400 μM final concentration) or DMSO as control for 60 min at 37°C, and then treated with 2 μM ActivX Desthiobiotin-FP probe (ThermoFisher Scientific) and processed as described above for *M*. *abscessus* R living cells. Detailed protocol regarding ABPP experiments is given in S1 Appendix.

### Mass spectrometry analysis for enzyme identification and quantification

Protein extract were loaded and stacked on a NuPAGE gel (Life Technologies). Stained bands were submitted to an in-gel trypsin digestion [19]. Peptides extracts were reconstituted with 0.1% trifluoroacetic acid in 4% acetonitrile and analyzed by liquid chromatography (LC)-tandem mass spectrometry (MS/MS) using Orbitrap Mass Spectrometers (Thermo Electron, Bremen, Germany) online with a nanoLC Ultimate 3000 chromatography system (Dionex, Sunnyvale, CA). Protein identification and quantification were processed using the MaxQuant computational proteomics platform, version 1.5.3.8 [20] using a UniProt *M*. *abscessus* ATCC 19977 (Taxon 561007) database (date 2017.02; 4940 entries). The statistical analysis was done with Perseus program (version 1.5.6.0). Differential proteins were detected using a two-sample *t*-test at 0.01 and 0.05 permutation-based FDR. Detailed Materials and Methods are given in S1 Appendix.

The mass spectrometry proteomics data have been deposited to the ProteomeXchange Consortium (www.proteomexchange.org) [21] *via* the PRIDE partner repository with the dataset identifier PXD015680.

### Validation of Ag85C_*Mabs*_ by iB*p*PPOX

#### Plasmids and DNA manipulations

All specific oligonucleotides and plasmids used in this study are listed in S1 Appendix (see S3 and S4 Tables—page S8). All cloned fragments were amplified using purified *M*. *abscessus* genomic DNA. The *mab_0175* gene encoding Ag85C was amplified by PCR using the specific forward (*pMyc*::*ag85C-F*) and reverse (*pMyc*::*ag85C-R*) primers. For the inactivated Ser124Ala mutant *ag85C*^*S124A*^ construction, overlap extension PCR (OE-PCR) was used. For the generation of first fragment containing the mutation of the active serine to alanine, primer sets *pMyc*::*ag85C-F* and *pMyc*::*ag85C*^*S124A*^*-R* were used, the second fragment containing the mutation was generated using the primer sets *pMyc*::*ag85C*^*S124A*^*-F and pMyc*::*ag85C-R*. The two fragments were further purified, mixed in 1:1 (*v/v*) ratio and used as template to amplify the complete insert containing the mutation, using the primer pairs *pMyc*::*ag85C-F* and *pMyc*::*ag85C-R*. The respective PCR products were cloned into pMyC vector, following digestion with NcoI and HindIII, enabling the incorporation of a _6_His-tag in the C-terminus of the Ag85C or Ag85C^S124A^ protein. Deletion mutant Δ*mab_0175* (= Δ*ag85C*) was obtained by a simple and rapid gene disruption strategy in *M*. *abscessus* developed by Viljoen *et al*. [22]. *Ag85C* gene was amplified using primer pairs *pUX1*::*Δag85C-F* and *pUX1*::*Δag85C-R*, then cloned into pUX1 vector using NheI and BamHI restriction sites by classical cloning. Finally, for complementation strain, the *mab_0175* gene was amplified using the primer pairs *pVV16*::*ag85C-F* and *pVV16*::*ag85C-R*, and cloned into pVV16 plasmid in frame with a _6_His-tag located in C-terminal and downstream of the *hsp60* promoter also containing a kanamycin resistance cassette using restriction free cloning (SLIC) [23] to generate *pVV16*::*ag85C*. Sequence integrity of each construct was confirmed by DNA sequencing (Eurofins Genomics). All the constructs were further transformed in electrocompetent *M*. *abscessus* S and R types and selected on respective antibiotic agar plates as described previously [22]. Positive transformants were further grown in 7H9^OADC^ medium (*i*.*e*., 7H9 broth + 0.2% glycerol + 0.05% Tween 80 + 10% oleic acid, albumin, dextrose, catalase) supplemented with either hygromycin (1000 μg/mL; *i*.*e*., overexpression and inactivated strains), kanamycin (250 μg/mL; *i*.*e*., deletion strain) or both antibiotics (1000 μg/mL hygromycin + 250 μg/mL kanamycin; *i*.*e*., complementation strain), up to OD_600_ of 1. The overproduction of the recombinant proteins in the overexpression and inactivated strains as well as in the complementation strain was checked by Western blot using the HisProbe^™^ HRP conjugate (ThermoFisher Scientific). Regarding the deletion strain, the selection was made based on red fluorescent colonies followed by PCR amplification and sequencing strategy as described in [22].

### Functional validation of Ag85C_*Mabs*_ target enzyme

The abovementioned transformed bacteria, *i*.*e*., the *M*. *abscessus*_pMyc::*ag85C* overexpressing strains, the inactivated *M*. *abscessus*_pMyc::*ag85C*^*S124A*^ overexpressing strains, the *M*. *abscessus*_Δ*ag85C* deletion strains and their complemented counterparts *M*. *abscessus*_Δ*ag85C*::C were grown in 7H9^OADC^ medium supplemented with either hygromycin (1000 μg/mL; *i*.*e*., overexpression and inactivated strains), kanamycin (250 μg/mL; *i*.*e*., deletion strain), or both antibiotics (1000 μg/mL hygromycin + 250 μg/mL kanamycin; *i*.*e*., complementation strain) until the OD_600_ reached 2. In the case of the overexpression and inactivated strains, induction was further done with 0.2% acetamide and the culture was incubated at 37°C for additional 24 h. Susceptibility testing of each of the *M*. *abscessus* mutant strains against various concentrations of **iB*p*PPOX** was further performed as described above.

### Expression and purification of *M*. *abscessus* antigen Ag85C

The plasmid harboring the *mab_0175* gene was used to transform the *M*. *smegmatis Δ*groEl expression strain. Transformed bacteria were grown in 7H9 medium containing hygromycin (200 μg/mL) until the OD_600_ reached 2.0. Induction was done with 0.2% acetamide and the culture was further incubated at 37 °C for 24 h. One L of bacterial pellets were collected by centrifugation (8,000 × *g*, 4 °C, 1 h), re-suspended in 30 mL ice-cold buffer (50 mM Tris pH 8.0 containing 200 mM NaCl), and were broken using a French Pressure cell at 1,100 psi. The lysate was clarified by centrifugation (12,000 × *g*, 4 °C, 30 min) prior to purification by nickel affinity chromatography with Ni-NTA sepharose beads and elution with the previous Tris (pH 8.0) buffer containing 500 mM imidazole. Purified protein was concentrated at 1 mg/mL and stored at ‒80 °C [24, 25].

### *In vitro* inhibition of pure recombinant *M*. *abscessus* Ag85C by iB*p*PPOX

A 14 μM (*i*.*e*., 25 μg) concentration of Ag85C_*Mabs*_ was incubated for 1 h in its native form with increasing molar excess of **iB*p*PPOX** (*i*.*e*. enzyme/inhibitor molar ratio, E/I = 1:1; 1:5, 1:10, 1:25, 1:50, and 1:75) in a reaction mixture containing 10 mM Tris buffer (pH 8), 150 mM NaCl and 0.1% (*w/v*) Triton X-100. Each sample was further treated with 10 μM ActiveX TAMRA-FP fluorescent probe (ThermoFisher Scientific) for 1 h at room temperature in the darkness. The reaction was stopped by adding 5X Laemmli reducing buffer followed by boiling, and equal amounts of proteins (12 μg) were separated by 12% SDS-PAGE. Subsequently, TAMRA FP-labeled proteins were detected by fluorescent gel scanning (TAMRA: λ_ex_ 557 nm, λ_em_ 583 nm) using the Cy^®^3 filter of a ChemiDoc MP Imager (Bio-Rad) before staining the gel with Coomassie Brilliant Blue dye. Finally, relative fluorescence quantification of each band was performed using the ImageLab^™^ software version 5.0 (Bio-Rad) by taking the labeled Ag85C_*Mabs*_-TAMRA adduct as 100% absolute fluorescence level.

### Mass spectrometry analysis of Ag85C_*Mabs*_-iB*p*PPOX complex

Purified Ag85C_*Mabs*_ recombinant protein (14 μM– 100 μg) was further incubated for 1 h in its native form with **iB*p*PPOX**, using an enzyme/inhibitor molar ratio E/I = 1:100 to ensure total inhibition. Samples of the resulting Ag85C_*Mabs*_-**iB*p*PPOX** complex were analysed on a MALDI-TOF-TOF Bruker Ultraflex III spectrometer (Bruker Daltonics, Wissembourg, France) controlled by the Flexcontrol 3.0 package (Build 51), as described previously [24] (see S1 Appendix for full details). The total mass of the untreated protein (theoretical Mw = 32,057.83 Da; experimental Mw = 32,048.7 Da) is corresponding to the native enzyme lacking the 36 first N-terminal amino acids (*i*.*e*., M^1^SVRVKARRVLSALLAAFVMPVSMAAAMTINPATAH^36^) consisting of a Sec signal peptide cleaved at the Ala-X-Ala (*i*.*e*., A^35^-H^36^-A^37^) site, as confirmed by N-terminal Edman sequencing [26].

### Statistical analysis

Graphpad Prism 5 was used to perform the statistical analyses of the intracellular activity of the **OX** compounds, and of all susceptibility testing on *M*. *abscessus* mutant strains. The statistical analysis related to MIC_50Raw_ was completed using a Student’s *t*-test. The statistical significance of differences in the MIC_50_ or MIC_90_ values between each mutant strain was analyzed by one-way ANOVA followed by a post hoc Fisher's test.

## Results and discussion

### *In vitro* activity of oxadiazolone derivatives against *M*. *abscessus*

Drug susceptibility testing of the **OX** derivatives was assessed against both S and R variants of *M*. *abscessus*, with amikacin (AMK) as standard drug. The corresponding MIC_50_/MIC_90_ values for each **OX** compound, as determined by the REMA assay [12–16], are reported in Table 1. Among all tested compounds, 14 **OXs** were able to block the growth of *M*. *abscessus* S variant. The best growth inhibitors were **iB*p*PPOX** (33.0 ±2.0 μM), **H*p*PPOX** (32.5 ±2.2 μM), **Me*m*PPOX** (41.8 ±1.6 μM) and **BePOX** (45.1 ±3.4 μM) which displayed interesting MIC_50_ values (Table 1). In all other cases, MIC_50_ values were indicative either of a moderate (MIC_50_ around 53–61 μM for **M*m*PPOX**, **iBPOX**, and **Be*p*PPOX**), a weak (MIC_50_ around 78–93 μM for **M*p*PPOX**, **E*m*PPOX**, **B*m*PPOX**, and **HPOX**), or a poor (MIC_50_ > 120 μM for **iB*m*PPOX**, **Eh*m*PPOX**, and **Be*m*PPOX**) antibacterial activity (Table 1). Considering the MIC_90_ values reached on *M*. *abscessus* S, they are up to 2.5-fold greater than the corresponding MIC_50_; except for **HPOX** (MIC_50_ = 92.9 ±4.2 μM / MIC_90_ = 99.9 ±5.5 μM), **Me*m*PPOX** (MIC_50_ = 41.8 ±1.6 μM / MIC_90_ = 44.4 ±2.0 μM) and **BePOX** (MIC_50_ = 45.1 ±3.4 μM / MIC_90_ = 46.5 ±2.0 μM) for which both MICs are in the same order of magnitude (Table 1).

**Table 1:** Antibacterial activities of the oxadiazolone derivatives against *M*. *abscessus* growth in broth medium using the REMA method^a^.

Compounds	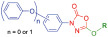	MIC_50_/MIC_90_ (μM)	
		*M*. *abscessus* CIP104536^T^	
		S variant	R variant
AMK		3.9 ±0.19 / 5.8 ±0.20	7.4 ±0.26 / 10.1 ±0.45
IMP		4.2 ±0.19 / 6.3 ±0.26	11.9 ±0.63 / 29.9 ±1.1
**M*m*PPOX**	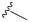	60.7 ±5.0 / 119.3 ±4.2	181 ±9.0 / >200
**M*p*PPOX**		88.2 ±7.3 / 157.5 ±6.2	>200
**MPOX**		>200	>200
**E*m*PPOX**	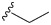	82.8 ±6.5 / 101.8 ±4.6	191.8 ±10.2 / >200
**Me*m*PPOX**	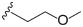	41.8 ±1.6 / 44.4 ±2.0	95.1 ±5.1 / 113.9 ±4.7
**B*m*PPOX**	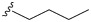	78.1 ±5.3 / >200	167.4 ±8.5 / 174.1 ±8.1
**iB*m*PPOX**	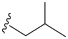	122.1 ±7.8 / >200	133.5 ±8.0 / >200
**iB*p*PPOX**		33.0 ±2.0 / 85.9 ±5.5	53.2 ±1.8 / 104.3 ±5.1
**iBPOX**		61.3 ±5.1 / 68.8 ±2.4	>200
**H*m*PPOX**	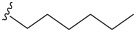	>200	120.3 ±7.1 / >200
**H*p*PPOX**		32.5 ±2.2 / 79.4 ±3.3	45.8 ±1.9 / 103.8 ±4.0
**HPOX**		92.9 ±4.2 / 99.9 ±5.5	>200
**Be*m*PPOX**	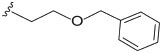	126.7 ±7.3 / 145.7 ±6.9	153.0 ±7.8 / >200
**Be*p*PPOX**		53.7 ±3.1 / 73.5 ±3.2	52.6 ±2.5 / 111.1 ±4.1
**BePOX**		45.1 ±3.4 / 46.5 ±2.0	98.0 ±5.8 / 170.2 ±6.2
**O*m*PPOX**	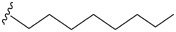	>200	135.9 ±6.7 / 150.9 ±5.5
**Eh*m*PPOX**	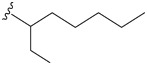	145.1 ±7.7 / >200	142.7 ±7.0 / 150.3 ±5.0
**D*m*PPOX**	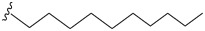	>200	144.0 ±7.8 / 167.5 ±5.8
**Do*m*PPOX**	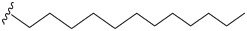	>200	104.6 ±5.2 / >200

^*a*^ Experiments were performed as described in **Materials and Methods**. MIC_50_ / MIC_90_: compound minimal concentration leading to 50% or 90% of growth inhibition, respectively, as determined by the REMA assay. Values are mean of at least two independent assays performed in triplicate. AMK, amikacin. IMP, imipenem.

Compared to the S morphotype, *M*. *abscessus* R variant was nearly 1.3- to 3.6-times less sensitive to the **OX** compounds (Table 1); a property already observed for many drugs including AMK [27]. The best inhibitors of *M*. *abscessus* R growth were **iB*p*PPOX** (MIC_50_ = 53.2 ±1.8 μM / MIC_90_ = 104.3 ±5.1 μM), **H*p*PPOX** (MIC_50_ = 45.8 ±1.9 μM / MIC_90_ = 103.8 ±4.0 μM), and **Be*p*PPOX** (MIC_50_ = 52.6 ±2.5 μM / MIC_90_ = 111.1 ±4.1 μM) which exhibited similar MIC_50_ and MIC_90_ values, respectively (Table 1). Interestingly, **M*p*PPOX** bearing a short methyl chain has no antibacterial effect as compared to the three abovementioned *para*-phenoxyphenyl derivatives. In summary, **iB*p*PPOX**, **H*p*PPOX**, and **Be*p*PPOX** all possessing the phenoxy group in a *para* position as well as bulky ester chains, displayed the best antibacterial activity against *M*. *abscessus* R. No other clear trends or rules in terms of structure-activity relationships (SAR) have emerged regarding the potency of these oxadiazolone-core compounds against *M*. *abscessus*.

It is noteworthy that with MIC_50_ values ranging from 31 to >120 μM [12], *M*. *tuberculosis* susceptibility to the **OX** compounds is similar to that of the S variant of *M*. *abscessus*; **iB*p*PPOX** being the best growth inhibitor of both species. The increased tolerance of the most-virulent *M*. *abscessus* R variant towards the **OX** compounds is in line with its high resistance to classical antibiotics [4] compared to *M*. *tuberculosis*; a result that supports *M*. *abscessus* R’s nickname of “antibiotics nightmare” [28].

### Intramacrophagic susceptibility of *Mycobacterium abscessus* to OX derivatives

Macrophages, as the primary target, represent the host's first line of defense but also an important reservoir of mycobacteria in lungs. From our previous work, the **OXs** were able to inhibit the growth of *M*. *tuberculosis* inside infected macrophages, and found to be non-toxic for Raw264.7 murine macrophages cell line with a CC_50_ > 100 μM (*i*.*e*., compound concentration leading to 50% cell toxicity) [12]. Considering such properties, we further investigated the ability of **OXs** to inhibit the intra-macrophagic growth of *M*. *abscessus*.

The intrinsic nature of the R variant is to form bacterial clumps and cords in culture medium with time. As reported by Bernut *et al*., *M*. *abscessus* R cording prevents its phagocytosis by macrophages. Consequently, the strain continues to grow extracellularly, and rapidly induces cell toxicity leading to cell death [29, 30]. Such cording characteristic makes macrophage infection experiments using *M*. *abscessus* R very difficult to handle. Indeed, nearly all macrophages were lysed at 24 h post-infection with *M*. *abscessus* R variant, making it impossible to quantify the intracellular effect of the **OXs**. This is, however, not the case with *M*. *abscessus* S for which more homogenous bacterial suspensions can be obtained for macrophages infection studies [25, 31, 32].

**Fig 2:**
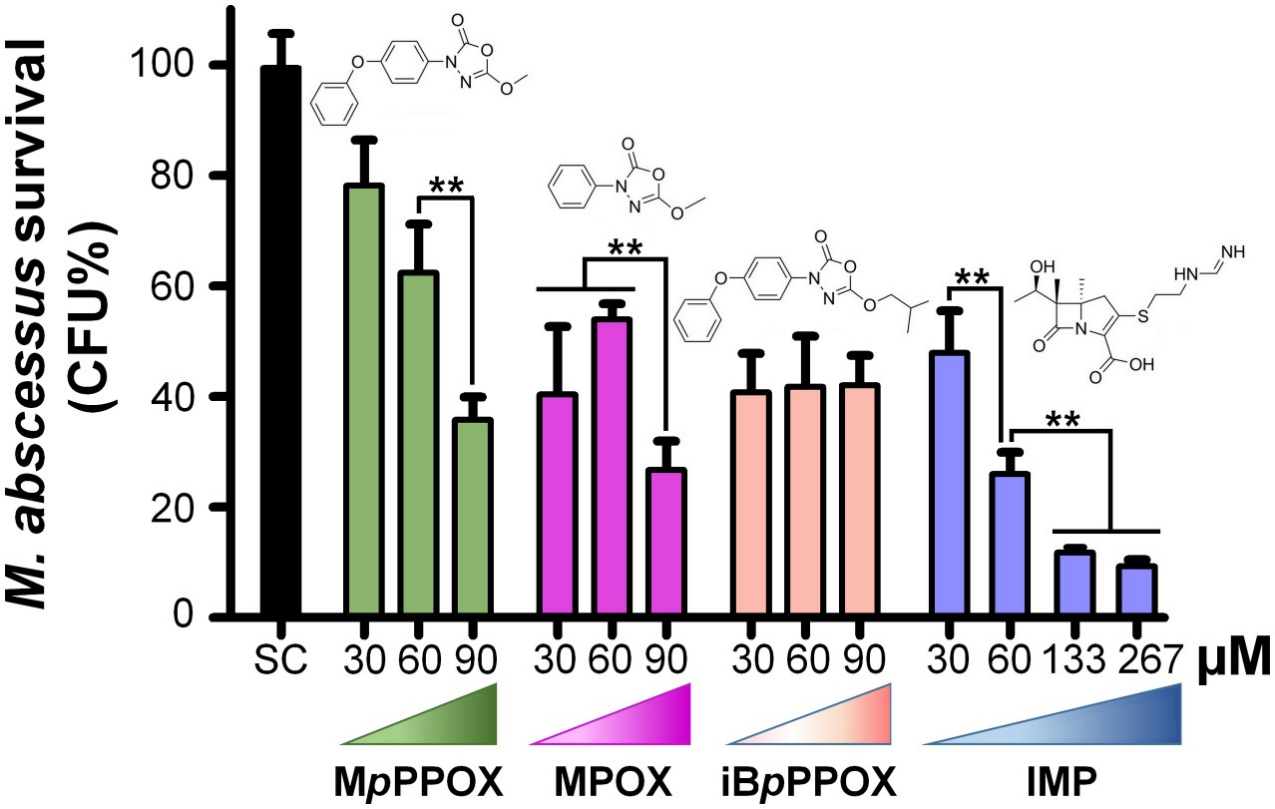
Intracellular activity of M*p*PPOX, MPOX and iB*p*PPOX as compared to imipenem (IMP). The activity of selected **OXs** on intracellular *M*. *abscessus* was tested in Raw264.7 murine macrophages. Cells were infected at a multiplicity of infection (MOI) of 10:1 with *M*. *abscessus* S variant and treated with various concentrations of each inhibitor or IMP for 24 h. Then, surviving bacteria were enumerated by plating serial dilutions of macrophage lysates. DMSO-treated infected macrophages (*i*.*e*., SC, solvent control) were used as control representing 100% of bacterial viability. Results are shown as mean ± standard error of the mean (SEM) of three independent assays performed in triplicate. **, *p*-value < 0.01. *, *p*-value < 0.05. Statistical analysis was done using a Student’s *t*-test.

Therefore, Raw264.7 cells were infected with *M*. *abscessus* S at a multiplicity of infection (MOI) of 10, and then incubated for 24 h with all the **OX** compounds at a final concentration of 90, 60 and 30 μM, or with imipenem (IMP) used as positive drug control. Among the 19 compounds tested, only 3 **OXs** (*i*.*e*., **MPOX**, **M*p*PPOX**, and **iB*p*PPOX**) exhibited an antibacterial activity against intracellular *M*. *abscessus* growth. Interestingly, **M*p*PPOX** and **MPOX**, which are weakly and not active against extracellular bacilli, respectively, were however able to significantly decrease the intramacrophagic *M*. *abscessu*s present 24 h after infection (Fig 2). **M*p*PPOX** displayed a moderate activity against intracellular *M*. *abscessus* S (Fig 2) with an approximated MIC_50Raw_ of around 75 μM which is 2.6 times higher that of IMP (MIC_50Raw_ = 28.3 μM). In contrast, 24 h-treatment with 30–60 μM **MPOX** led to a 53% reduction in mycobacteria which increased up to 73.5% at 90 μM, a percentage value comparable to the one elicited by IMP, *i*.*e*., 74.0% reduction following treatment with 60 μM (Fig 2). Remarkably, and as observed previously for *M*. *tuberculosis* [12], **iB*p*PPOX** was the sole identified inhibitor able to impair extracellular as well as intracellular growth of *M*. *abscessus*. A plateau value corresponding to 58.5 ±0.8% bacterial killing was indeed reached, whatever the **iB*p*PPOX** concentration used (30–90 μM) to treat the infected cells.

Such a difference between the intra and extracellular activities has already been reported in our previous works with the **OX** derivatives [12], as well as with another family of growth inhibitors, the Cyclipostins & Cyclophostin analogs [13, 14] acting against *M*. *tuberculosis* and *M*. *abscessus* [25]. Similar to *M*. *tuberculosis* [12], the intracellular and extracellular inhibition of *M*. *abscessus* growth may probably result from several different mechanisms of action or penetration of the **OX** derivatives. The short methyl chain **M*p*PPOX** and **MPOX** display a better antimycobacterial activity against intramacrophagic *M*. *abscessus* than in broth medium. This clear preference against intracellularly-replicating mycobacteria may imply that the intracellular activity and/or the targets of these two compounds might differ from that of **OXs** acting on extracellularly-replicating bacilli. Several factors may indeed account for these discrepancies, such as the metabolic status/fitness which varies between extra- and intracellular replicating bacteria. Another hypothesis could be that their corresponding target(s) would be more accessible and/or vulnerable during the intracellular lifestyle of *M*. *abscessus*. A specific response of the macrophage stimulated by the action of these compounds and leading to bacterial clearance cannot, however, be excluded. On the other hand, the **iB*p*PPOX** retains a similar activity against *M*. *abscessus* both extracellularly (MIC_50_ = 33.0 μM) and inside macrophages (~59% bacterial clearance at 30 μM). Regarding its intracellular antibacterial activity, the presence of a plateau value, whatever the concentration used, might underline a different effect of **iB*p*PPOX** towards infected macrophages compared to **M*m*PPOX** and **MPOX** for which a more classical dose-response has been reached. As mentioned above, one can speculate that the cellular stress caused by the action of **iB*p*PPOX** on the infected macrophages might induce a specific stringent response of these host cells, such as possible cell metabolism, therefore leading to bacterial death.

Given the previously determined very low toxicity of the three selected compounds toward Raw264.7 cells with CC_50_ > 100 μM [12] similar to AMK (CC_50_ ≥ 150 μM) [33], the selectivity index (SI = CC_50_/MIC_50Raw_) of these best intracellular inhibitors on *M*. *abscessus vs*. Raw264.7 cells was thus valued to be in a range from around 1.3 for **M*p*PPOX** and up to >3 for **iB*p*PPOX**.

From these findings, it can be assumed that the observed inhibitory potency of the **OX** compounds *i*) might result from the inhibition of specific but most likely distinct mycobacterial target enzymes between intramacrophagic- *vs*. extracellularly-replicating bacilli; or *ii*) may reflect differences in the uptake and accumulation of the different compound inside the macrophage. Overall, these results suggest that both **M*p*PPOX**, **MPOX** and **iB*p*PPOX** would be able to enter the macrophages and arrest bacterial replication without exhibiting significant toxicity for the host cell.

### iB*p*PPOX inhibit *M*. *abscessus* by targeting various serine/cysteine enzymes

Given the previous results obtained with the **HPOX** on target enzymes identification during *M*. *tuberculosis in vitro* growth in broth medium [12], we thus performed a similar ABPP approach [12, 13, 34–37] to identify the potential target enzymes impacted by **iB*p*PPOX**, the sole extra and intracellular inhibitor of *M*. *abscessus* growth.

The R variant being associated to the most virulent form of *M*. *abscessus* and thus to severe pulmonary infections [6, 28, 38]; a crude lysate of *M*. *abscessus* R was, in the first approach, incubated with the **iB*p*PPOX** inhibitor (or DMSO as a control) and then subjected to competitive probe labelling/enrichment assay with the ActivX^™^ Desthiobiotin-FP probe (ThermoFisher Scientific), as reported previously in the case of *M*. *tuberculosis* [12, 13]. The obtained enriched mixtures were further digested with trypsin, and the resulting peptides were analyzed by liquid chromatography-tandem mass spectrometry (LC-MS/MS) followed by subsequent label free quantification analysis. The proteins also found in the control experiment (*i*.*e*., DMSO alone for unspecific binding to streptavidin-magnetic beads) were not considered. A panel of 58 distinct protein candidates were then identified with a permutation false discovery rate (pFDR) of 10%, which was reduced to 21 and 11 when applying a pFDR of 5% and 1%, respectively (see S1 Table).

Since most of the identified proteins were putative in *M*. *abscessus*, the corresponding orthologs in *M*. *tuberculosis* H37Rv have been reported to bring more information about their essentiality, activity and predicted location [39]. Eleven out of 21 identified proteins (at a pFDR of 5%) were (Ser/Cys)-based enzymes, mainly involved in lipid metabolism and cell wall biosynthesis [40, 41]. These included the probable serine protease PepD (MAB_1078); the D-amino acid aminohydrolase MAB_2605c (*i*.*e*., Rv2913c); the probable carboxylesterase MAB_1919 (*i*.*e*., Rv2223c); and the putative β-lactamase MAB_2833 (*i*.*e*., Rv1367c) possibly involved in cell wall biosynthesis. Three members of the lipase family Lip [42], LipH (MAB_2039), LipN (MAB_3270c) and LipI (MAB_2814); three Cutinase-like proteins [41], Cut2 (MAB_3263), Cut3 (MAB_3765) and Cut4 (MAB_3766); and MAB_175 (Ag85C), a member of the antigen 85 (Ag85) complex [24, 43] which catalyzes the biosynthesis of trehalose dimycolate, triacylglycerol as well as the mycolylation of arabinogalactan, were also uncovered with **iB*p*PPOX**.

In a second approach, similar ABPP experiments were performed on living bacterial cells in order to take into account the ability of **iB*p*PPOX** to penetrate/diffuse through the mycobacterial cell wall. Accordingly, *M*. *abscessus* R cells were grown to log phase and incubated with **iB*p*PPOX** or DMSO as a control. After cell lysis, the obtained total lysate was processed as described above with ActivX^™^ Desthiobiotin-FP probe and streptavidin magnetic beads. Tryptic digestion followed by tandem mass spectrometry analysis led to the identification of 21 protein candidates at a pFDR of 5%, and only 5 at a pFDR of 1% (Table 2 and S2 Table).

**Table 2:** iB*p*PPOX target proteins identified at a pFDR of 1% and 5% in *M*. *abscessus* R culture by LC-ESI-MS/MS analysis.

Protein Ids	Mol. Weight [kDa]	*M*. *tuberculosis* orthologs				
		Rv number	Essentiality ^a^	Location ^b^	Activity / Function	Functional category ^c^
**MAB_0176**	**35.825**	**Rv3804c**		**CF/M**	**Secreted antigen 85-A FbpA (Ag85A)**	**LM**
**MAB_0177**	**34.909**	**Rv3804c**		**CF/M/WCL**	**Antigen 85-A/B/C precursor**	**LM**
MAB_0274c	20.371			-	*uncharacterized protein*	-
MAB_0401	46.209	Rv0517		-	Possible acyltransferase	IM/R
MAB_0520	38.811	Rv3626c		-	*uncharacterized protein*	-
**MAB_0684c**	**26.813**	**Rv0774c**		**CF**	**Hypothetical extracellular esterase**	**CW/CP**
MAB_1053c	10.305	Rv0948c	*In vitro growth*	WCL	Chorismate mutase	IM/R
MAB_1675	28.418	Rv2362c	*In vitro growth*	CW	Possible DNA repair protein RecO	IP
MAB_2366	33.804	Rv1701		-	Probable integrase	RP
MAB_2477c	55.217	Rv1393c		-	Probable monoxygenase	IM/R
MAB_2478c	15.382			-	*uncharacterized protein*	-
MAB_2545c	35.436	Rv0480c		M/WCL	Possible amidohydrolase	IM/R
MAB_2943c	31.546	Rv1543		M/WCL	Possible fatty acyl-CoA reductase	LM
**MAB_3336c**	**54.339**	**Rv2045c**		**-**	**Carboxylesterase LipT**	**IM/R**
MAB_3398	17.635	Rv3178		-	*uncharacterized protein*	-
MAB_3661	57.093	Rv3308		M	Probable phosphomannomutase PmmB	IM/R
**MAB_3689**	**26.374**	**Rv3342**		**WCL**	**Possible methyltransferase**	**IM/R**
MAB_3705	19.995	Rv2506		CF/M	Putative TetR family regulatory protein	RP
MAB_4103c	30.192	Rv1523		-	Probable methyltransferase	IM/R
MAB_4201c	22.905	Rv3574		WCL	Transcriptional regulatory protein KstR	RP
MAB_4750	27.932	Rv1544		M/WCL	Possible ketoacyl reductase	LM

In **bold**, the 5 proteins identified at a pFDR of 1%.

^*a*^ From [44, 45].

^*b*^ CF: Culture filtrate; CW: Cell wall; M: Membrane fraction; WCL: Whole cell lysate.

^*c*^ IM/R: intermediary metabolism/respiration; IP: information pathways; CW/CP: cell wall/cell processes; LM: lipid metabolism; RP: regulatory protein.

Although 4 of the identified proteins are only conserved hypotheticals, the remaining 17 ranged in their functional category from intermediary metabolism/respiration (8 proteins), lipid metabolism (4 proteins), regulatory pathways (3 proteins), cell wall/cell processes (1 protein), and information pathways (1 protein). Among them, MAB_1675, the probable DNA repair protein RecO (*i*.*e*., Rv2362c), and MAB_1053c (*i*.*e*., Rv0948c) a putative chorismate mutase possibly involved in phenylalanine, tyrosine and tryptophan biosynthesis, are annotated as essential enzymes for the *in vitro* growth of *M*. *tuberculosis* [44, 45]. In good agreement with our previous work on *M*. *tuberculosis* target enzymes [12], several hydrolases were detected, including one hypothetical extracellular esterase (MAB_2181c), three putative methyltransferases (MAB_3689, MAB_4103c, MAB_0401); the carboxylesterase LipT (MAB_3336c) belonging to the Lip-family members, and the mycolyltransferases MAB_176 (Ag85A) and MAB_177 (Ag85-A/B/C precursor) two members of the Ag85 complex (Table 2 and S2 Table).

It is noteworthy that among these 21 potential hits, only Ag85 proteins were previously detected in the **iB*p*PPOX**-treated total lysate (see S1 and S2 Tables); thus, implying that nearly 19 proteins had not been detected in the previous treated *M*. *abscessus* total lysate, or at least at a pFDR ≤ 10%. On the other hand, such result suggests that Antigen 85 proteins may be the first target enzymes encountered and thus inhibited by the **OX** compounds.

### Validation of M. abscessus Ag85C as vulnerable target of iBpPPOX

Knowing the importance of the Ag85 complex in mycobacterial membrane integrity due to its central role in cell envelope biogenesis, and given the fact that inhibiting the Ag85C was found to restrict *M*. *tuberculosis* growth [46], we decided to confirm the Ag85C_*Mabs*_, which shares nearly 58% amino acid sequence identity with its *M*. *tuberculosis* ortholog and retains the same conserved catalytic triad (*i*.*e*., Ser^124^-Glu^228^-His^260^), as a potential target of the **OX** compounds.

We thus followed two different strategies: the first one was based on the susceptibility testing of various *M*. *abscessus* mutant strains to the **iB*p*PPOX**; and the second one relied on the molecular interaction between the **iB*p*PPOX** and the purified recombinant Ag85C_*Mabs*_.

**Fig 3:**
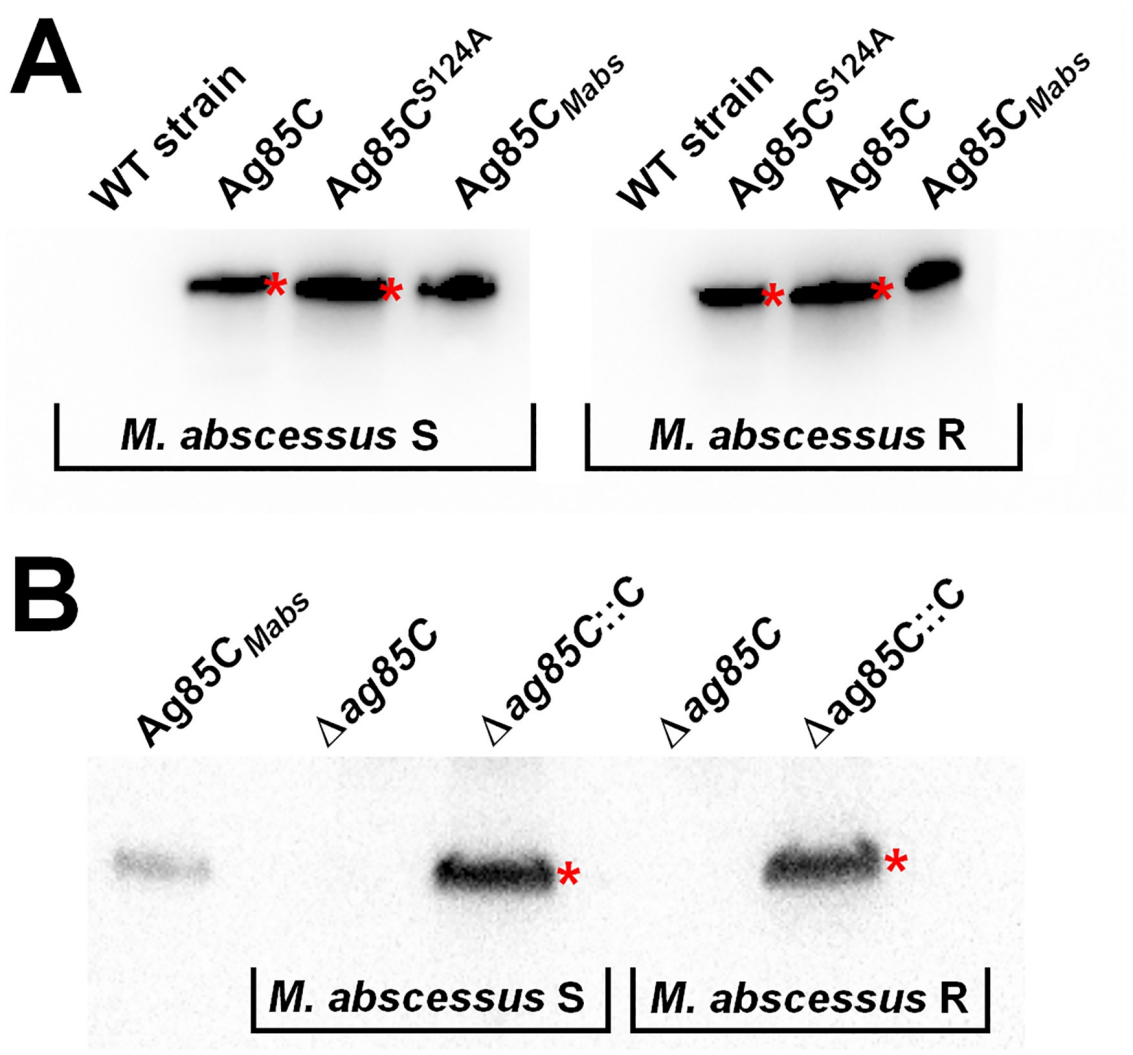
Western blot analysis of *M*. *abscessus*-Ag85C-mutant strains: (A) overexpression of active (*i*.*e*., Ag85C) or inactivated (*i*.*e*., Ag85C^S124A^) protein; (B) deletion (*i*.*e*., Δ*ag85C*) and complementation (*i*.*e*., Δ*ag85C*::C) strains (see S1 Appendix file for cloning details). Each overexpressed protein, indicated with a red star, were revealed using HisProbe^™^ HRP conjugate (Thermo-Fisher Scientific) and compared to the *M*. *abscessus* wild-type strain (WT) as well as pure recombinant Ag85C_*Mabs*_ protein, as control. In each case, equal amount of whole bacterial cell lysate has been loaded for the overexpression strains (panel **A**), and for the deletion/complementation strains (panel **B**), respectively.

In the first step, genes encoding either Ag85C_*Mabs*_ or the inactivated Ag85C^S124A^ protein were cloned and overexpressed in *M*. *abscessus* S and R variants using the pMyC::*ag85C* / pMyC::*ag85C*^*S124A*^ inducible plasmids, where genes were cloned under the control of an acetamide promoter (Fig 3A). Moreover, a deletion mutant of Ag85C_*Mabs*_ named Δ*ag85C* was generated by using a recent one-step single cross-over system with the pUX1 vector [22]; and its complemented counterpart Δ*ag85C*::C (Fig 3B) was obtained using the pVV16::*ag85C* complementation plasmid which allows the constitutive production of recombinant Ag85C_*Mabs*_ under the control of the *hsp60* promoter (see S1 Appendix for cloning details). In each case, the overexpression/complementation of antigen 85C protein was confirmed by Western blotting as compared to the parental strain (WT) (Fig 3).

In order to examine whether the overexpression, inactivation or deletion/complementation of the Ag85C_*Mabs*_ protein affect the strain susceptibility to the **iB*p*PPOX** compound, their respective MICs were further determined.

As depicted in Table 3, the overexpression of Ag85C_*Mabs*_ protein (*i*.*e*., *M*. *abscessus* S_pMyc::*ag85C* and *M*. *abscessus* R_pMyc::*ag85C*) led to a significant increase in MIC_50_ values by 2.7-fold for both the S (87.3 ±3.4 μM; *p*-value <0.01) and R variant (148.2 ±2.1 μM; *p*-value <0.01), as well as in MIC_90_ values (>200 μM), compared to the respective pMyc vector control and wild-type strains. These results clearly suggest that Ag85C_*Mabs*_ is responsible for the decreased susceptibility to the **iB*p*PPOX**, thus confirming this protein as one of the targets of our compound.

**Table 3:** Variation of MIC (μM) of iB*p*PPOX against *M*. *abscessus*-Ag85C-mutant strains^a^.

*M*. *abscessus* strains	MIC_50_ / MIC_90_ (μM)	MIC_50_ / MIC_90_ ratio mutant *vs*. WT
*M*. *abscessus* S WT	33.0 ±2.0^**¶**^ / 85.9 ±5.5^**┴**^	1.0 / 1.0
*M*. *abscessus* S_pMyc empty vector	31.9 ±1.7 / 82.4 ±0.92	0.97 / 0.96
*M*. *abscessus* S_pMyc::*ag85C*^*S124A*^	34.4 ±3.0 / 83.1 ±6.8	1.04 / 0.97
*M*. *abscessus* S_Δ*ag85C*	33.7 ±1.9 / 81.5 ±7.4	1.02 / 0.95
*M*. *abscessus* S_Δ*ag85C*::C	32.6 ±1.3 / 87.4 ±1.5	0.99 / 1.02
***M*. *abscessus* S_pMyc::*ag85C***	**87.3 ±3.4**^**¶**^ **/ >200**^**┴**^	**2.65 / >3.0**
*M*. *abscessus* R WT	53.2 ±1.8^**‡,†,§**^ / 104.3 ±5.1^**║**^	1.0 / 1.0
*M*. *abscessus* R_pMyc empty vector	49.9 ±2.6 / 109.2 ±10.4	0.94 / 1.05
*M*. *abscessus* R_pMyc::*ag85C*^*S124A*^	47.5 ±2.0^**‡**,^* / 119.0 ±9.6	0.89 / 1.14
*M*. *abscessus* R_Δ*ag85C*	30.9 ±2.1^**†**,^*,^**#**^ / 114.9 ±8.2	0.58 / 1.10
*M*. *abscessus* R_Δ*ag85C*::C	51.8 ±3.1^**#**^ / 108.2 ±4.6	0.97 / 1.04
***M*. *abscessus* R_pMyc::*ag85C***	**148.2 ±2.1**^**§**^ **/ >200**^**║**^	**2.78 / >2**

^*a*^ Experiments were performed as described in **Materials and Methods**. MIC_50_ / MIC_90_: compound minimal concentration leading to 50% or 90% growth inhibition, respectively. Values are mean of two independent assays performed in triplicate. MIC values with a common symbol are significantly different (^**‡**^: *p*-value<0.05; ^**¶, ┴, †, §, ║**^, *, ^**#**^: *p*-value<0.01; ANOVA followed by Fisher’s test).

Regarding the inactivated Ag85C^S124A^ mutant *M*. *abscessus* S_pMyc::*ag85C*^*S124A*^, the gene deletion mutant *M*. *abscessus* S_Δ*ag85C* and its complemented counterpart *M*. *abscessus* S_Δ*ag85C*::C, as well as the wild-type *M*. *abscessus* S strain, they all responded similarly to **iB*p*PPOX**. In the case of *M*. *abscessus* R, although no significant variation was observed in MIC_90_ values (mean MIC_90_ = 111.1 ±8.4 μM), a slight decrease in MIC_50_ of around 0.89- to 0.58-fold was reached for the inactivated Ag85C^S124A^ (47.5 ±2.0 μM; *p*-value <0.05) and the Δ*ag85C* (30.9 ±2.1 μM; *p*-value <0.01) mutants, respectively, compared to the wild-type strain (53.2 ±1.8 μM); while complementation of Ag85C_*Mabs*_ (*i*.*e*., *M*. *abscessus* R_Δ*ag85C*::C) restored the wild-type R phenotype (51.8 ±3.1 μM—Table 3).

**Fig 4:**
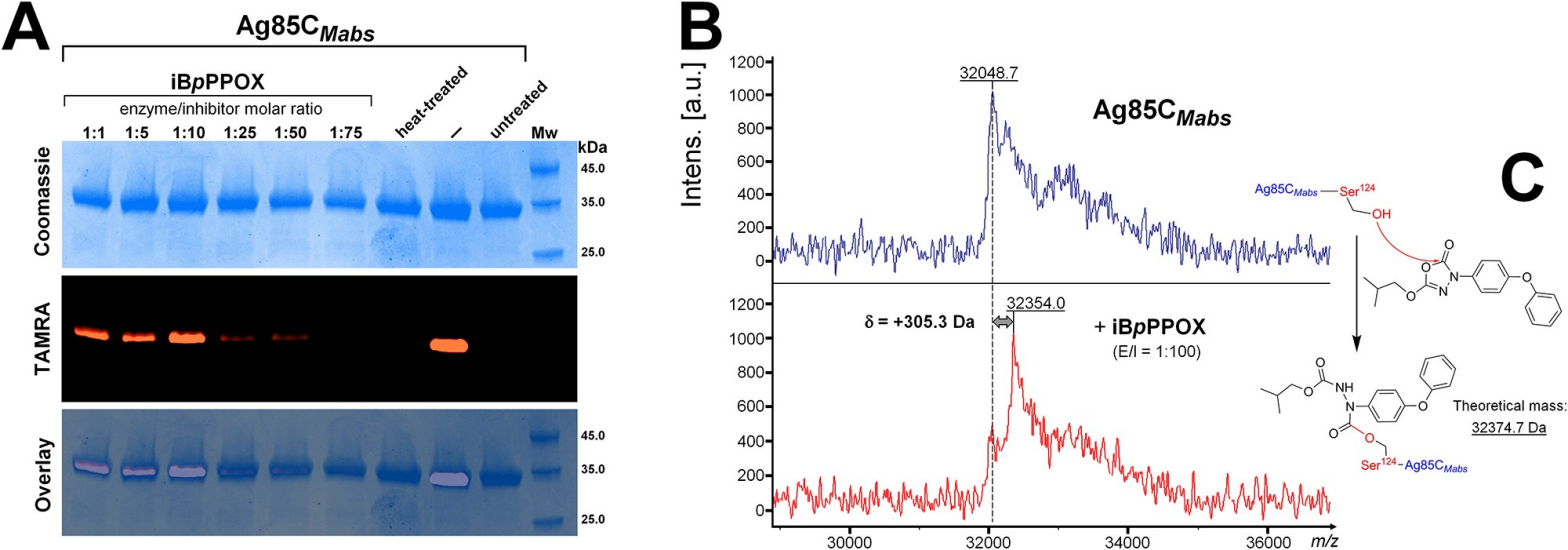
Inhibition of the Ag85C_*Mabs*_ by iB*p*PPOX. (A) Ag85C_*Mabs*_ was pre-treated with **iB*p*PPOX** (*i*.*e*. enzyme/inhibitor molar ratio of 1:1 to 1:75), incubated with ActiveX TAMRA-FP, separated by 12% SDS-PAGE, and visualized by Coomassie blue staining (*upper panel*) or in-gel fluorescence visualization (*middle panel*). The merged image is shown in the *lower panel*. Untreated protein (*i*.*e*., no TAMRA-FP and no **iB*p*PPOX**) was used as control. No TAMRA-FP labeling is detected in the presence of inactivated heat-treated Ag85C_*Mabs*_. TAMRA labeling of Ag85C_*Mabs*_ is impaired in the Ag85C_*Mabs*_-**iB*p*PPOX** adducts, as evidenced by the loss of fluorescence in the **iB*p*PPOX** lanes, presumably resulting from the covalent binding of **iB*p*PPOX** to the catalytic serine as previously observed [24, 25]. TAMRA-labeled Ag85C_*Mabs*_ was detected by fluorescent gel scanning (λ_ex_ 557 nm, λ_em_ 583 nm) using the Cy^®^3 filter of a ChemiDoc MP Imager (Bio-Rad) before staining of the gel with Coomassie Brilliant Blue dye. Relative fluorescence quantification of each band was performed using the ImageLab^™^ software version 5.0 (Bio-Rad) by taking as 100% absolute fluorescence level the labeled Ag85C_*Mabs*_-TAMRA adduct. (B) Global mass modification of Ag85C_*Mabs*_ pre-incubated with **iB*p*PPOX**, at an enzyme/inhibitor molar ratio of 1:100 to ensure total inhibition, as determined using an MALDI-TOF-TOF mass spectrometer in linear mode. (C) Mechanism of inhibition of Ag85C_*Mabs*_ by the oxadiazolone **iB*p*PPOX**, based on mass spectrometry analysis. a.u., arbitrary units.

Based on these results, purified Ag85C_*Mabs*_ recombinant protein [25] was further incubated with **iB*p*PPOX**, using increasing enzyme/inhibitor molar ratio (E/I) ranging from 1:1 to 1:75, and then treated with ActivX TAMRA-FP fluorescent probe, as reported previously [24, 25]. Equal amounts of proteins were separated on SDS-PAGE and visualized by Coomassie staining or in-gel fluorescence for TAMRA detection (Fig 4A). Relative fluorescence quantification of each band was done using the ImageLab^™^ software version 5.0 (Bio-Rad) by taking as 100% absolute fluorescence level, the labeled Ag85C_*Mabs*_-TAMRA adduct (Fig 4A). As expected, pre-treating Ag85C_*Mabs*_ with **iB*p*PPOX**, resulted in a significant loss in fluorescence intensity by around 32.8 ±1.8% (E/I = 1:1 to 1:10), 58.5 ±0.70% (E/I = 1:25), 64.0 ±1.8% (E/I = 1:50) and up to >90% (E/I = 1:75) as compared to the non-treated protein labeled by the TAMRA-FP probe (Fig 4A). This means that the TAMRA-FP probe cannot bind the catalytic serine when the Ag85C_*Mabs*_-**iB*p*PPOX** complex has been formed, as revealed by the significant loss in fluorescence emission (Fig 4A).

MALDI-TOF mass spectrometry was further used to confirm the (covalent) nature of the inhibition. Sample of the Ag85C_*Mabs*_-**iB*p*PPOX** (E/I = 1:100) complex was subjected to MALDI-TOF mass spectrometry analyses. Mass increment of +305.3 Da was then observed within the global mass of the inhibited Ag85C_*Mabs*_ as compared with the untreated protein (Fig 4B); whereas no changes in the global mass were observed with the inactivated heat-treated protein. Such result is thus consistent with the formation of a covalent enzyme-inhibitor adduct, as the reaction between the catalytic Ser124 and **iB*p*PPOX** is expected to yield a mass increase of +326 Da; and also, in agreement with the mechanism of action of such **OX** derivatives [42]. All these findings conclusively indicate that pure recombinant Ag85C_*Mabs*_ protein is covalently modified by the **iB*p*PPOX** derivative (Fig 4C), in good agreement with the known classical mechanism of action of such **OX** compounds as previously demonstrated using pure lipolytic enzymes [12, 42].

Taken together, the *in vitro* inhibitory experiments conducted with **iB*p*PPOX** on pure recombinant Ag85C_*Mabs*_ protein (Fig 4), as well as the statistically significant increased resistance levels when overexpressing the Ag85C_*Mabs*_ protein in *M*. *abscessus* S and R variants (Table 3), thus confirm the assertion that this enzyme is an effective target of **iB*p*PPOX**.

## Conclusion

As already highlighted in the case of *M*. *tuberculosis* [12], our series of oxadiazolone-core **OX** derivatives are able to impair different metabolic pathways during either extracellular and/or intracellular bacterial growth *via* the inhibition of various (Ser/Cys)-based enzymes, therefore resulting in *M*. *abscessus* death. Although the efficiency of these **OX** molecules could not be considered as sufficient enough to obtain powerful anti-mycobacterial agents, they may however represent attractive tools for deciphering the lipid metabolism in *M*. *abscessus* and/or in *M*. *tuberculosis*. We have indeed reported that the **M*m*PPOX** compound was able to prevent intracytoplasmic lipid inclusion (ILI) catabolism *in vivo* in *M*. *bovis* BCG infected murine bone-marrow-derived macrophages (mBMDM) [47–49]; as well as *in vitro* under carbon excess and nitrogen-deprived conditions allowing ILI biosynthesis and hydrolysis in *M*. *abscessus* [50]. Taken together, all these findings support that the **OX** derivatives are able to abolish the activity of several (Ser/Cys)-containing enzymes involved in mycobacterial lipid metabolism and/or in cell wall biosynthesis. This is the case of the Ag85 complex proteins which are essential players in the biosynthesis of lipids from mycobacterial membrane as well as in intracellular lipid metabolism, but also of proteins belonging to the hormone-sensitive lipase (HSL) family member proteins (*i*.*e*., Lip-HSL) [42], including LipY the major Lip-HSL lipase involved in mycobacterial lipid catabolism [49–52]. Therefore, the respective effects of these **OX** compounds against lipid-poor *vs*. lipid-rich bacteria deserve to be investigated in more details. More especially, deciphering how the presence of intracytoplasmic lipid inclusions (ILI) in lipid-rich bacteria can actively contribute to substantially enhanced mycobacterial virulence and pathogenesis as compared to lipid-poor strains, as reported recently [50], will provide major insights for understanding the general development of mycobacterial-related diseases. Such experiments are currently underway, and will be reported in due course.

## Supporting information

S1 Appendix Detailed protocols regarding the MIC determination, targets identification and mass spectrometry analysis of Ag85C_*Mabs*_; as well as the list of plasmids and primers used in this study. (PDF)

S1 Fig Uncropped and unadjusted image for Western Blotting of Fig 3. Each overexpressed protein was revealed using the HisProbe^™^ HRP conjugate (ThermoFisher Scientific) and compared to the *M*. *abscessus* wild type strain as well as the pure recombinant Ag85C_*Mabs*_ protein.
(TIF)

S2 Fig Uncropped and unadjusted images for SDS-PAGE gel of Fig 4A. SDS-PAGE gel visualized by Coomassie blue staining (*upper panel*) or by in-gel fluorescence visualization (*middle panel*). Superimposition of both images is reported in the *lower panel*. Molecular weights were derived from the Unstained Protein Molecular Weight Marker (Euromedex).
(TIF)

S1 Table iBpPPOX target proteins identified in M. abscessus R total lysate by LC-ESI-MS/MS analysis. Only positive hits with a pFDR of 1%, 5% and 10% are reported.
(XLSX)

S2 Table iBpPPOX target proteins identified in M. abscessus R culture cell by LC-ESI-MS/MS analysis. Only positive hits with a pFDR of 1% and 5% are reported.
(XLSX)

## Abbreviations

ABPP: activity-based protein profiling
AMK: amikacin
CC_50_: compound concentration leading to 50% Raw264.7 macrophages toxicity
CF: cystic fibrosis
CFU: colony-forming units
IMP: imipenem
MIC_50_ / MIC_90_: minimal inhibitory concentration leading to 50% or 90% bacterial growth inhibition, respectively
NTM: non-tuberculous mycobacteria
OX: oxadiazolone
pFDR: permutation false discovery rate
REMA: resazurin microtiter assay
