# Supplementary material for: Dissecting the antibacterial activity of oxadiazolone-core derivatives against Mycobacterium abscessus
Source: PLoS One. 2020 Sep 18;15(9):e0238178. doi: 10.1371/journal.pone.0238178 (PMC7500638; doi:10.1371/journal.pone.0238178)
Supplement: S1 Appendix — (PDF) [file pone.0238178.s001.pdf]

# Supplemental Material

## Dissecting the antibacterial activity of Oxadiazolone-core derivatives against *Mycobacterium abscessus*

Abdeldjalil Madani[1¶], Ivy Mallick[1,2¶], Alexandre Guy[3], Céline Crauste[3], Thierry Durand[3], Patrick Fourquet[4], Stéphane Audebert[4], Luc Camoin[4], Stéphane Canaan[1], and Jean-François Cavalier[1*]

[1] Aix-Marseille Univ., CNRS, LISM, Institut de Microbiologie de la Méditerranée, Marseille, France
[2] IHU Méditerranée Infection, Aix-Marseille Univ., Marseille, France
[3] IBMM, Univ Montpellier, CNRS, ENSCM, Montpellier, France
[4] Aix-Marseille Univ, INSERM, CNRS, Institut Paoli-Calmettes, CRCM, Marseille Protéomique, Marseille, France

*** Corresponding author**
E-mail: jfcavalier@imm.cnrs.fr

[¶] These authors contributed equally to this work and should be considered as first coauthors.

**Contents**

Detailed protocols for susceptibility testing, ABPP experiments and mass spectrometry analysis of Ag85C*Mabs* S2

S3 and S4 Tables: primers and plasmids, respectively used in this study S8

References S9

**Resazurin microtiter assay (REMA) for MIC determination.** Susceptibility testing was performed using the Middlebrook 7H9 broth microdilution method. MICs of the **OXs** were determined in 96-well flat-bottom Nunclon Delta Surface microplates with lid (Thermo-Fisher Scientific, ref. 167008) using the resazurin microtiter assay (REMA) [1-3]. Briefly, log-phase bacteria were diluted to a cell density of 5 × $10^6$ cells/mL in 7H9-S (7H9 broth + 0.2% glycerol + 0.05% Tween 80 + 0.2% glucose). Then 100 µL of the above inoculum (*i.e.*, 5 × $10^5$ cells per well) was added to each well containing 100 µL 7H9-S medium, serial two-fold dilutions of the **OX** analogs or controls to a final volume of 200 µL. Growth controls containing no inhibitor (*i.e.*, bacteria only = *B*), inhibition controls containing 50 µg/mL kanamycin and sterility controls (*i.e.*, medium only = *M*) without inoculation were also included. Plates were incubated at 37 °C in a humidity chamber [4] to prevent evaporation for 3-5 days. Then, 20 µL of a 0.025% (*w/v*) resazurin solution was added to each well, and the plates were incubated at 37 °C for color change from blue to pink or violet and for a reading of fluorescence units (FU). Fluorescence corresponding to the resazurin reduction to its metabolite resorufin was quantified using a Tecan Spark 10M multimode microplate reader (Tecan Group Ltd, France) with excitation at 530 nm and emission at 590 nm. For fluorometric MIC determinations, a background subtraction was performed on all wells with a mean of *M* wells. Relative fluorescence units were defined as: RFU% = (test well FU/mean FU of *B* wells) × 100. MIC values were determined by fitting the RFU% sigmoidal dose-response curves [2, 3] in Kaleidagraph 4.2 software (Synergy Software). The lowest compound concentration inhibiting 50% and 90% of bacterial growth was defined as the $MIC_{50}$ and $MIC_{90}$, respectively.

**Intramacrophage killing assay.** The intracellular growth of *M. abscessus* S was assessed following a 24 h exposure of infected Raw264.7 murine macrophages cell line (American Type Culture Collection TIB-71) to each of the 19 **OX** compounds at a final concentration of 30 µM [5]. Murine (Raw264.7) macrophages were grown in Dulbecco's modified Eagle medium (DMEM; Gibco) supplemented with 10% heat-inactivated fetal calf serum (FBS, Invitrogen) ($DMEM^{FBS}$) medium at 37 °C and 5% $CO_2$ to subconfluent concentrations. Then 5 × $10^4$ cells/well were seeded in 96-well flat-bottom Nunclon Delta Surface microplates with lid (ThermoFisher Scientific, ref. 167008) in a final volume of 200 µL per well, and cultured for additional 24 h. The cells were infected with *M. abscessus* S at a multiplicity of infection (MOI) of 1:10 and incubated at 37 °C in the presence of 5% $CO_2$ for 3 h. Cells were then washed three times with DMEM then refed with $DMEM^{FBS}$ supplemented with 200 µg/mL amikacin (340 µM; 87 × $MIC_{50}$) for 1 h. at 37 °C and 5% $CO_2$ to kill all extra-cellular bacteria; washed again three times with DMEM prior to the addition of 2-fold dilutions of **OX** compounds or imipenem (IMP; 80 µg/mL = 267 µM; 24 × $MIC_{50}$) in $DMEM^{FBS}$ supplemented with 50 µg/mL (*i.e.*, 85.4 µM) amikacin (200 µL final volume). In each plate, negative

controls consisting of amikacin (50 µg/mL) and 1% DMSO (*i.e.*, infected macrophages only); as well as positive controls containing amikacin (50 µg/mL) plus 80 µg/mL (*i.e.*, 267 µM) IMP were also included. Plates were incubated for 24 h at 37 °C, 5% $CO_2$. In each case, the viability of infected macrophages was checked by addition of trypan blue [6] before cell lysis. Cells were washed three times with PBS and lysed by adding 200 µL of 0.1% Triton X-100. Serial dilutions of each culture were then plated at least in triplicate on 7H9 agar medium supplemented with 0.2% glucose. Colonies were counted after 4 to 5 days of incubation at 37 °C to check intracellular bacterial viability following treatment with each compound concentration. DMSO-treated infected macrophages corresponded as control representing 100% of bacterial viability. Intracellular $MIC_{Raw}$ values were estimated by fitting the CFU% sigmoidal dose-response curves in Kaleidagraph 4.2 software (Synergy Software). The lowest compound concentration inhibiting 50% of intracellular bacterial growth was defined as the $MIC_{50Raw}$. Experiments were done three times independently.

**Activity-Based Protein Profiling (ABPP) for iB*p*PPOX target enzymes identification.** From 300 mL of culture at the logarithmically growth stage ($OD_{600}$ ~1.5), *M. abscessus* R cells were harvested by centrifugation at 4,000 *g* for 15 min, and adjusted to a final concentration of $6 \times 10^9$ cells/mL (*i.e.*, $OD_{600}$ of 40) in 7H9-S (7H9 broth + 0.2% glycerol + 0.05% Tween 80 + 0.2% glucose). One mL of this suspension was incubated with **iB*p*PPOX** (400 µM final concentration), or DMSO (control) at 37 °C for 2-3 h. under gentle shaking at 75 rpm. Bacteria were then washed 3 times with PBS containing 0.05% Tween 80, and resuspended in PBS buffer at a 1:1 (*w/v*) ratio. The bacterial cells (500 µL) were mixed with 350 µL of 0.1 mm diameter glass beads (BioSpec) in a 2-mL Eppendorf tube and disrupted during 2 × 4 min of violent shaking, with ice cooling between each run, using mini-Beadbeater-96 (BioSpec). The lysate was cooled down in ice for 5 min and then centrifuged at 4°C and at 13,500 *g* for 15 min to remove the cell debris and unbroken cells. Supernatants were adjusted to a concentration of 1 mg/mL of total proteins, snap frozen in liquid nitrogen and stored at –80°C until further use.

Both **iB*p*PPOX**-treated *M. abscessus* R and DMSO-control lysate samples (750 µL – 0.75 mg total proteins) were incubated with 2 µM ActivX™ Desthiobiotin-FP probe for 90 min at room temperature. The reaction was next stopped by adding 0.45 g of urea (10 M final concentration) to complete denaturation of proteins. Unreacted probes were removed using Zeba Spin desalting column (7K MWCO, ThermoFisher Scientific) and labelled proteins were further captured by 200 µg Nanolink streptavidin magnetic beads 0.8 µm (Solulink), according to the manufacturer's instructions. First, 20 µL of a 10 mg/mL NanoLink streptavidin magnetic beads was transferred into a 1.5 mL Eppendorf tube. The Wash Buffer (50 mM Tris-HCl, 150 mM NaCl, 0.05% Tween 20, pH 8.0) was then added to bring the final volume to 250 µL and the resulting mixture was mixed gently

to resuspend and wash the beads. The tube was placed on a magnetic stand for 2 min. and the supernatant was discarded. The tube was removed from the magnetic stand and the beads were washed two more times with the Wash Buffer (250 µL). Each *M. abscessus* treated-lysate sample was enriched for labelled proteins by transfer to the previously washed beads (around 200 µg). The lysate/beads suspensions were incubated for 1 h. at room temperature with mild shaking. The tubes were then placed on the magnetic stand for 2 min to collect the beads, and the supernatant was removed. The beads containing bound, biotinylated proteins were washed three times carefully with the Wash Buffer, as described above, and resuspended in 30 µL PBS buffer pH 7.4 containing 50 mM free biotin. The resulting solution was mixed with 4X Laemmli reducing sample buffer, and heated at 95°C for 5 min. This step allowed the recovery of the captured labelled proteins by exchanging the initially captured desthiobiotin/streptavidin complex to the greater affinity biotin/streptavidin complex. Each sample was snap frozen in liquid nitrogen and stored at –80°C before mass spectrometry experiments.

To check for unspecific binding, a DMSO-treated lysate sample was also incubated only with the streptavidin-magnetic beads in absence of Desthiobiotin-FP probe treatment, and processed as described above.

**Capture of *M. abscessus* potential target enzymes from iB*p*PPOX-treated total lysate *via* ABPP experiments.** From 1 L of culture at the logarithmically growth stage (OD600 ~1), *M. abscessus R* cells were harvested by centrifugation at 4,000 × *g* for 15 min. Pellets were washed 3 times with PBS containing 0.05% Tween 80. The cell pellets were resuspended in PBS buffer at a 1:1 (*w/v*) ratio. The bacterial cells were then mixed with the same volume of 0.1 mm diameter glass beads (BioSpec) and disrupted during 4 min of violent shaking using Mini-Beadbeater-96 (BioSpec). Total lysate was adjusted to a concentration of 2 mg/mL of total proteins, snap frozen in liquid nitrogen and stored at –80 °C until further use. *M. abscessus* R lysates (500 µL – 1 mg total proteins) were further pre-incubated with **iB*p*PPOX** (400 µM final concentration), or DMSO as control for 60 min at 37 °C, and then treated with 2 µM ActivX Desthiobiotin-FP probe (ThermoFisher Scientific) and processed as described above for *M. abscessus* R living cells.

**Mass spectrometry analysis.** Protein extract were loaded on NuPAGE 4-12% Bis-Tris acrylamide gels (Life Technologies) to stack proteins in a single band that was stained with Imperial Blue (Pierce, Rockford, IL) and cut from the gel. Gels pieces were submitted to an in-gel trypsin digestion [7] with slight modifications. Briefly, gel pieces were washed and destained using 100 mM $NH_4HCO_3$. Destained gel pieces were shrunk with 100 mM ammonium bicarbonate in 50% acetonitrile and dried at room temperature. Protein spots were then rehydrated using 100 mM ammonium bicarbonate pH

8.0 buffer containing 10 mM DTT for 45 min at 56 C. This solution was replaced by 100 mM ammonium bicarbonate pH 8.0 buffer containing 55 mM iodoacetamide and the gel pieces were incubated for 30 min at room temperature in the dark. They were then washed twice in 100 mM ammonium bicarbonate buffer and finally shrunk by incubation for 5 min with 100 mM ammonium bicarbonate buffer in 50% acetonitrile. The resulting alkylated gel pieces were dried at room temperature. The dried gel pieces were re-swollen by incubation in same buffer supplemented with trypsin (12.5 ng/µL; Promega) for 1 h at 4 °C and then incubated overnight at 37 °C. Peptides were harvested by collecting the initial digestion solution and carrying out two extractions; first in 5% formic acid and then in 5% formic acid in 60% acetonitrile. Pooled extracts were dried down in a centrifugal vacuum system. Samples were reconstituted with 0.1% trifluoroacetic acid in 4% acetonitrile and analyzed by liquid chromatography (LC)-tandem mass spectrometry (MS/MS) using an LTQ-Orbitrap Velos Mass Spectrometer (Thermo Electron, Bremen, Germany) for *M. abscessus* treated-lysates, or using an Orbitrap Fusion Lumos Tribrid Mass Spectrometer (Thermo Electron, Bremen, Germany) for *M. abscessus* treated-cells, both online with a nanoLC Ultimate 3000 chromatography system (Dionex, Sunnyvale, CA). Peptides were separated on a Dionex Acclaim PepMap RSLC C18 column. First peptides were concentrated and purified on a pre-column from Dionex (C18 PepMap100, 2 cm × 100 µm I.D, 100 Å pore size, 5 µm particle size) in solvent A (0.1% formic acid in 2% acetonitrile). In the second step, peptides were separated on a reverse phase LC EASY-Spray C18 column from Dionex (PepMap RSLC C18, 15 or 50 cm × 75 µm I.D, 100 Å pore size, 2 µm particle size) at 300 nL/min flow rate. After column equilibration using 4% of solvent B (20% water - 80% acetonitrile - 0.1% formic acid), peptides were eluted from the analytical column by a two steps linear gradient (4-20% acetonitrile/$H_2O$; 0.1% formic acid for 90 min and 20-45% acetonitrile/$H_2O$; 0.1% formic acid for 30 min).

For peptide ionization (*i.e.*, samples of *M. abscessus* R treated-cells) in the EASY-Spray nanosource in front of the Orbitrap Fusion Lumos Tribrid Mass Spectrometer, spray voltage was set at 2.2 kV and the capillary temperature at 275 °C. The Orbitrap Lumos was used in data dependent mode to switch consistently between MS and MS/MS. Time between Masters Scans was set to 3 sec. MS spectra were acquired with the Orbitrap in the range of *m/z* 400-1,600 at a FWHM resolution of 120,000 measured at 400 *m/z*. AGC target was set at 4.0e5 with a 50 ms Maximum Injection Time. For internal mass calibration the 445.120025 ion was used as lock mass. The more abundant precursor ions were selected and collision-induced dissociation fragmentation was performed in the ion trap to have maximum sensitivity and yield a maximum amount of MS/MS data. Number of precursor ions was automatically defined along run in 3 sec windows using the "Inject Ions for All Available pararallelizable time option" with a maximum injection time of 300 ms. The signal threshold for an MS/MS event was set to 5,000 counts. Charge state screening was enabled to exclude precursors with

0 and 1 charge states. Dynamic exclusion was enabled with a repeat count of 1 and duration of 60 sec.

For peptide ionization (*i.e.*, samples of *M. abscessus* R treated-total lysates) in the EASY-Spray nanosource in front of the LTQ-Orbitrap Velos, spray voltage was set at 1.9 kV and the capillary temperature at 275 °C. The Orbitrap Velos was used in data dependent mode to switch consistently between MS and MS/MS. Time between Masters MS spectra were acquired with the Orbitrap in the range of *m/z* 300-1700 at a FWHM resolution of 30,000 measured at 400 *m/z*. For internal mass calibration the 445.120025 ion was used as lock mass. The more abundant precursor ions were selected and collision-induced dissociation fragmentation was performed in the ion trap on the 10 most intense precursor ions measured to have maximum sensitivity and yield a maximum amount of MS/MS data. The signal threshold for an MS/MS event was set to 5,000 counts. Charge state screening was enabled to exclude precursors with 0 and 1 charge states. Dynamic exclusion was enabled with a repeat count of 1, exclusion list size 500 and exclusion duration of 30 sec.

**Protein identification and quantification**. Relative intensity-based label-free quantification (LFQ) was processed using the MaxLFQ algorithm [8] from the freely available MaxQuant computational proteomics platform, version 1.5.3.8 [9]. The acquired raw LC Orbitrap MS data were first processed using the integrated Andromeda search engine [10]. Spectra were searched against a UniProt *M. abscessus* ATCC 19977 (Taxon 561007) database (date 2,017.02; 4,940 entries). This database was supplemented with a set of 245 frequently observed contaminants. The following parameters were used for searches: (***i***) trypsin allowing cleavage before proline; (***ii***) two missed cleavages were allowed; (***iii***) monoisotopic precursor tolerance of 20 ppm in the first search used for recalibration, followed by 4.5 ppm for the main search and 0.5 Da for fragment ions from MS/MS ; (***iv***) cysteine carbamidomethylation (+57.02146) as a fixed modification and methionine oxidation (+15.99491) and N-terminal acetylation (+42.0106) as variable modifications; (***v***) a maximum of five modifications per peptide allowed; and (***vi***) minimum peptide length was 7 amino acids and a maximum mass of 4,600 Da. The match between runs option was enabled to transfer identifications across different LC-MS/MS replicates based on their masses and retention time within a match time window of 0.7 min and using an alignment time window of 20 min. The quantification was performed using a minimum ratio count of 1 (unique+razor) and the second peptide option to allow identification of two co-fragmented co-eluting peptides with similar masses. The false discovery rate (FDR) at the peptide and protein levels were set to 1% and determined by searching a reverse database to limit the list of identified proteins. All proteins that cannot be distinguished based on their identified peptides were assembled into a single protein group according to the MaxQuant rules. The statistical analysis was done with Perseus program (version 1.5.6.0) from the MaxQuant environment

(www.maxquant.org). The LFQ normalised intensities were uploaded from the proteinGroups.txt file. First, proteins marked as contaminant, reverse hits, and "only identified by site" were discarded. Quantifiable proteins were defined as those detected in at least 100% of samples in at least one condition. Protein LFQ normalized intensities were base 2 logarithmized to obtain a normal distribution. Missing values were replaced using data imputation by randomly selecting from a normal distribution centred on the lower edge of the intensity values that simulates signals of low abundant proteins using default parameters (a downshift of 1.8 standard deviation and a width of 0.3 of the original distribution). In this way, imputation of missing values in the controls allows statistical comparison of protein abundances that are present only in the inhibitor's samples. To determine whether a given detected protein was specifically differential a two-sample *t*-test was done using permutation-based FDR-controlled at 0.01 and 0.05, and employing 250 permutations. The *p* value was adjusted using a scaling factor s0 with a value of 1 [11].
The mass spectrometry proteomics data have been deposited to the ProteomeXchange Consortium (www.proteomexchange.org) [12] *via* the PRIDE partner repository with the dataset identifier PXD015680.

**Mass spectrometry analysis of Ag85C*Mabs*-iB*p*PPOX complex.** Purified Ag85C*Mabs* recombinant protein (14 µM – 100 µg) was further incubated for 1 h in its native form with **iB*p*PPOX**, using an enzyme/inhibitor molar ratio E/I= 1:100 to ensure total inhibition. Samples of the resulting Ag85C*Mabs*-**iB*p*PPOX** complex were analysed on a MALDI-TOF-TOF Bruker Ultraflex III spectrometer (Bruker Daltonics, Wissembourg, France) controlled by the Flexcontrol 3.0 package (Build 51), as described previously [13]. This instrument was used at a maximum accelerating potential of 25 kV and was operated in linear mode using the *m/z* range from 20,000 to 100,000 (LP_66 kDa Method). Five external standards (Protein Calibration Standard II, Bruker Daltonics) were used to calibrate each spectrum to a mass accuracy within 200 ppm. Peak picking was performed with Flexanalysis 3.0 software (Bruker) with an adapted analysis method. To eliminate salts from the samples, 10 µL of each preparation was submitted to a desalting step on a C4 Zip-Tip µcolumn (Millipore). 1 µL of desalted sample was mixed with 1 µL α-cyano-4-hydoxycinnamic acid matrix in a 50% acetonitrile/0.3% TFA mixture (1:1, *v/v*). 1 µL was spotted on the target, dried and analysed with the LP_66 kDa method. Peak picking was performed with Flexanalysis 3.0 software (Bruker) with an adapted analysis method. Parameters used were as follows: SNAP peak detection algorithm, S/N threshold fixed to 6 and a quality factor threshold of 30.

**S3 Table: Primers used in this study. Restriction sites if present are underlined.**

| Gene | Primer | Sequence (5’-3’) | Restriction site |
|---|---|---|---|
| *pMyc::ag85C-F* | Forward | 5’–TAA<u>CCATGG</u>TTAGCGTGCGCGTGAAAGCCCG–3’ | NcoI |
| *pMyc::ag85C-R* | Reverse | 5’–GC<u>AAGCTT</u>TGCCGTTAAGCGTGGCAATCAGATC–3’ | HindIII |
| *pMyc::ag85C$^{S124A}$-F* | Forward | 5’-GTCGTCGGGCTGGCGATGAGTGGCG-3’ | |
| *pMyc::ag85C$^{S124A}$-R* | Reverse | 5’-CGCCACTCATCGCCAGCCCGACGAC-3’ | |
| *pUX1::Δag85C-F* | Forward | 5’-CCAACTTGTCTGGTGT<u>GCTAGC</u>TTGAGCGTGCGCGTGAAAGCCC-3’ | NheI |
| *pUX1::Δag85C-R* | Reverse | 5’-GCGTACACCGCCGCGGGCTAA<u>GGATCC</u>AGCTGCAGAATTCG-3’ | BamHI |
| *pVV16::ag85C-F* | Forward | 5’-GGAATCACTTC<u>CATATG</u>AGCGTGCGCGTGAAAGCCCG-3’ | NdeI |
| *pVV16::ag85C-R* | Reverse | 5’-GTGTGGTGGTGGTG<u>AAGCTT</u>GCCGTTAAGCGTGGCAATCAGATC-3’ | HindIII |

**S4 Table: Plasmids used in this study.**

| Name | Description | Reference |
|---|---|---|
| pMyc | Multicopy *E. coli* - mycobacterium shuttle vector, hygromycin cassette, acetamide inducible promoter | [14] |
| pUX1 | Plasmid produced by ligating the colE1 origin and kanamycin cassettes-containing XmnI fragment of pMV261-AflII to the blunted SpeI fragment from pTEC27 containing the tdTomato and hygromycin cassettes. | [15] |
| pVV16 | Multicopy *E. coli* - mycobacterium shuttle vector, kanamycin cassette, *hsp60* constitutive promoter | [14] |

## References


1. Palomino JC, Martin A, Camacho M, Guerra H, Swings J, Portaels F. Resazurin microtiter assay plate: simple and inexpensive method for detection of drug resistance in *Mycobacterium tuberculosis*. Antimicrob Agents Chemother. 2002;46(8):2720-2. Epub 2002/07/18. doi: https://doi.org/10.1128/aac.46.8.2720-2722.2002. PubMed PMID: 12121966; PubMed Central PMCID: PMC127336.

2. Rybniker J, Vocat A, Sala C, Busso P, Pojer F, Benjak A, et al. Lansoprazole is an antituberculous prodrug targeting cytochrome bc1. Nat Commun. 2015;6:7659. Epub 2015/07/15. doi: https://doi.org/10.1038/ncomms8659. PubMed PMID: 26158909; PubMed Central PMCID: PMC4510652.

3. Nguyen PC, Delorme V, Bénarouche A, Martin BP, Paudel R, Gnawali GR, et al. Cyclipostins and Cyclophostin analogs as promising compounds in the fight against tuberculosis. Scientific Reports. 2017;7(1):11751. doi: https://doi.org/10.1038/s41598-017-11843-4.

4. Walzl A, Kramer N, Mazza MR, Falkenhagen D, Hengstschläger M, Schwanzer-Pfeiffer D, et al. A Simple and Cost Efficient Method to Avoid Unequal Evaporation in Cellular Screening Assays, Which Restores Cellular Metabolic Activity. Int J Appl Sci Technol. 2012;2(6):17–21.

5. Rodrigues Felix C, Gupta R, Geden S, Roberts J, Winder P, Pomponi SA, et al. Selective Killing of Dormant *Mycobacterium tuberculosis* by Marine Natural Products. Antimicrob Agents Chemother. 2017;61(8):e00743-17. doi: https://doi.org/10.1128/aac.00743-17.

6. Strober W. Trypan blue exclusion test of cell viability. Curr Protoc Immunol. 2001;Appendix 3(1):Appendix 3B. Epub 2008/04/25. doi: https://doi.org/10.1002/0471142735.ima03bs21. PubMed PMID: 18432654.

7. Shevchenko A, Wilm M, Vorm O, Mann M. Mass spectrometric sequencing of proteins silver-stained polyacrylamide gels. Anal Chem. 1996;68(5):850-8. Epub 1996/03/01. doi: https://doi.org/10.1021/ac950914h. PubMed PMID: 8779443.

8. Cox J, Hein MY, Luber CA, Paron I, Nagaraj N, Mann M. Accurate proteome-wide label-free quantification by delayed normalization and maximal peptide ratio extraction, termed MaxLFQ. Mol Cell Proteomics. 2014;13(9):2513-26. Epub 2014/06/20. doi: https://doi.org/10.1074/mcp.M113.031591. PubMed PMID: 24942700; PubMed Central PMCID: PMC4159666.

9. Cox J, Mann M. MaxQuant enables high peptide identification rates, individualized p.p.b.-range mass accuracies and proteome-wide protein quantification. Nat Biotechnol. 2008;26(12):1367-72. Epub 2008/11/26. doi: https://doi.org/10.1038/nbt.1511. PubMed PMID: 19029910.

10. Cox J, Neuhauser N, Michalski A, Scheltema RA, Olsen JV, Mann M. Andromeda: a peptide search engine integrated into the MaxQuant environment. J Proteome Res. 2011;10(4):1794-805. Epub 2011/01/25. doi: https://doi.org/10.1021/pr101065j. PubMed PMID: 21254760.

11. Tusher VG, Tibshirani R, Chu G. Significance analysis of microarrays applied to the ionizing radiation response. Proc Natl Acad Sci U S A. 2001;98(9):5116-21. Epub 2001/04/20. doi: https://doi.org/10.1073/pnas.091062498. PubMed PMID: 11309499; PubMed Central PMCID: PMCPMC33173.

12. Vizcaino JA, Deutsch EW, Wang R, Csordas A, Reisinger F, Rios D, et al. ProteomeXchange provides globally coordinated proteomics data submission and dissemination. Nat Biotechnol. 2014;32(3):223-6. Epub 2014/04/15. doi: https://doi.org/10.1038/nbt.2839. PubMed PMID: 24727771; PubMed Central PMCID: PMC3986813.

13. Viljoen A, Richard M, Nguyen PC, Fourquet P, Camoin L, Paudal RR, et al. Cyclipostins and Cyclophostin analogs inhibit the antigen 85C from *Mycobacterium tuberculosis* both *in vitro* and *in vivo*. J Biol Chem. 2018;293(8):2755–69. doi: https://doi.org/10.1074/jbc.RA117.000760.

14. Santucci P, Point V, Poncin I, Guy A, Crauste C, Serveau-Avesque C, et al. LipG a bifunctional phospholipase/thioesterase involved in mycobacterial envelope remodeling. Bioscience Reports. 2018;38(6):BSR20181953. doi: https://doi.org/10.1042/bsr20181953.

15. Viljoen A, Gutierrez AV, Dupont C, Ghigo E, Kremer L. A Simple and Rapid Gene Disruption Strategy in Mycobacterium abscessus: On the Design and Application of Glycopeptidolipid Mutants. Front Cell Infect Microbiol. 2018;8:69. Epub 2018/03/30. doi: https://doi.org/10.3389/fcimb.2018.00069. PubMed PMID: 29594066; PubMed Central PMCID: PMCPMC5861769.
