# Supplementary figures and images for: Dissecting the antibacterial activity of oxadiazolone-core derivatives against Mycobacterium abscessus
Source: PLoS One. 2020 Sep 18;15(9):e0238178. doi: 10.1371/journal.pone.0238178 (PMC7500638; doi:10.1371/journal.pone.0238178)

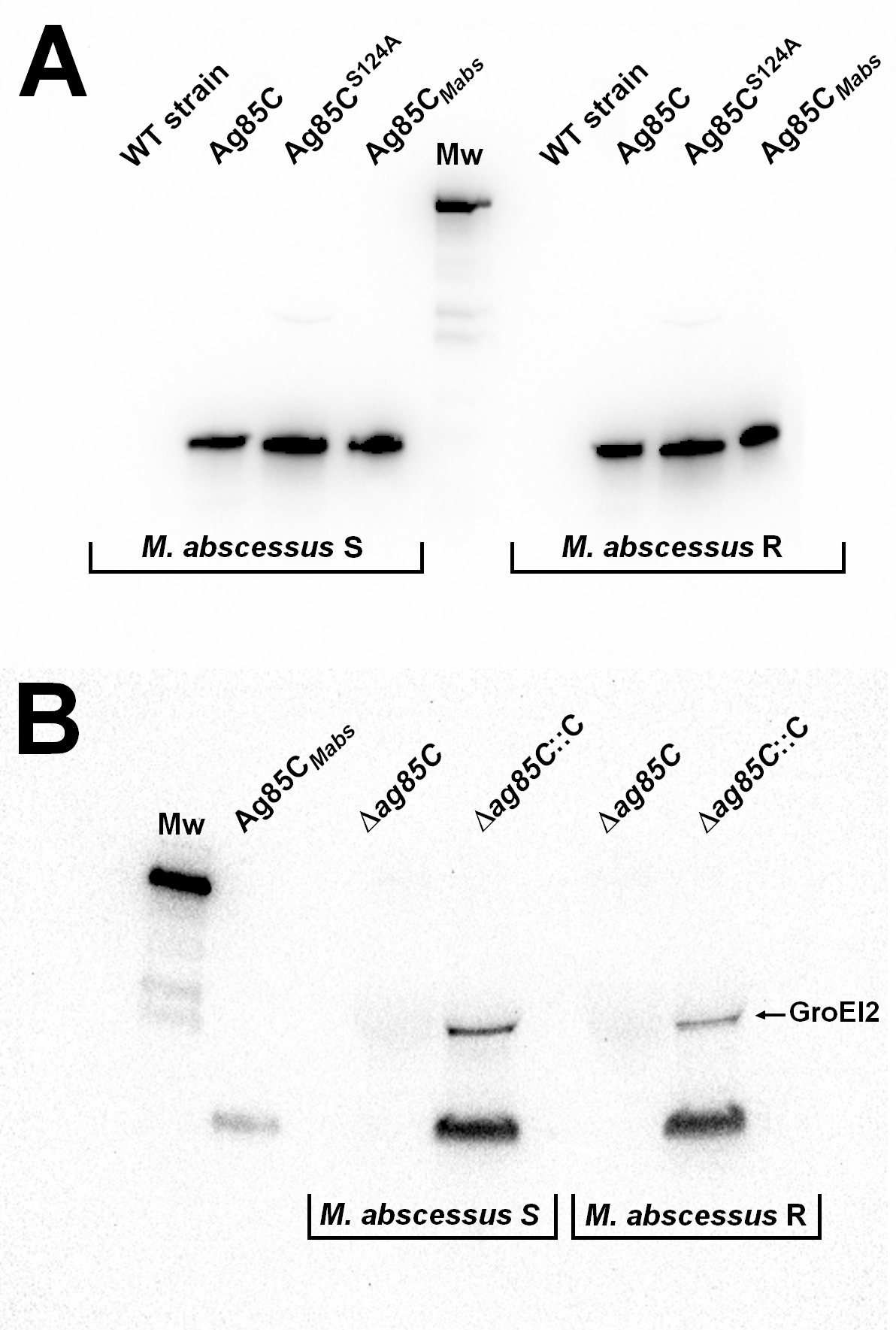

Supplement: S1 Fig — Each overexpressed protein was revealed using the HisProbe™ HRP conjugate (ThermoFisher Scientific) and compared to the M. abscessus wild type strain as well as the pure recombinant Ag85CMabs protein. (TIF) [file pone.0238178.s002.tif]

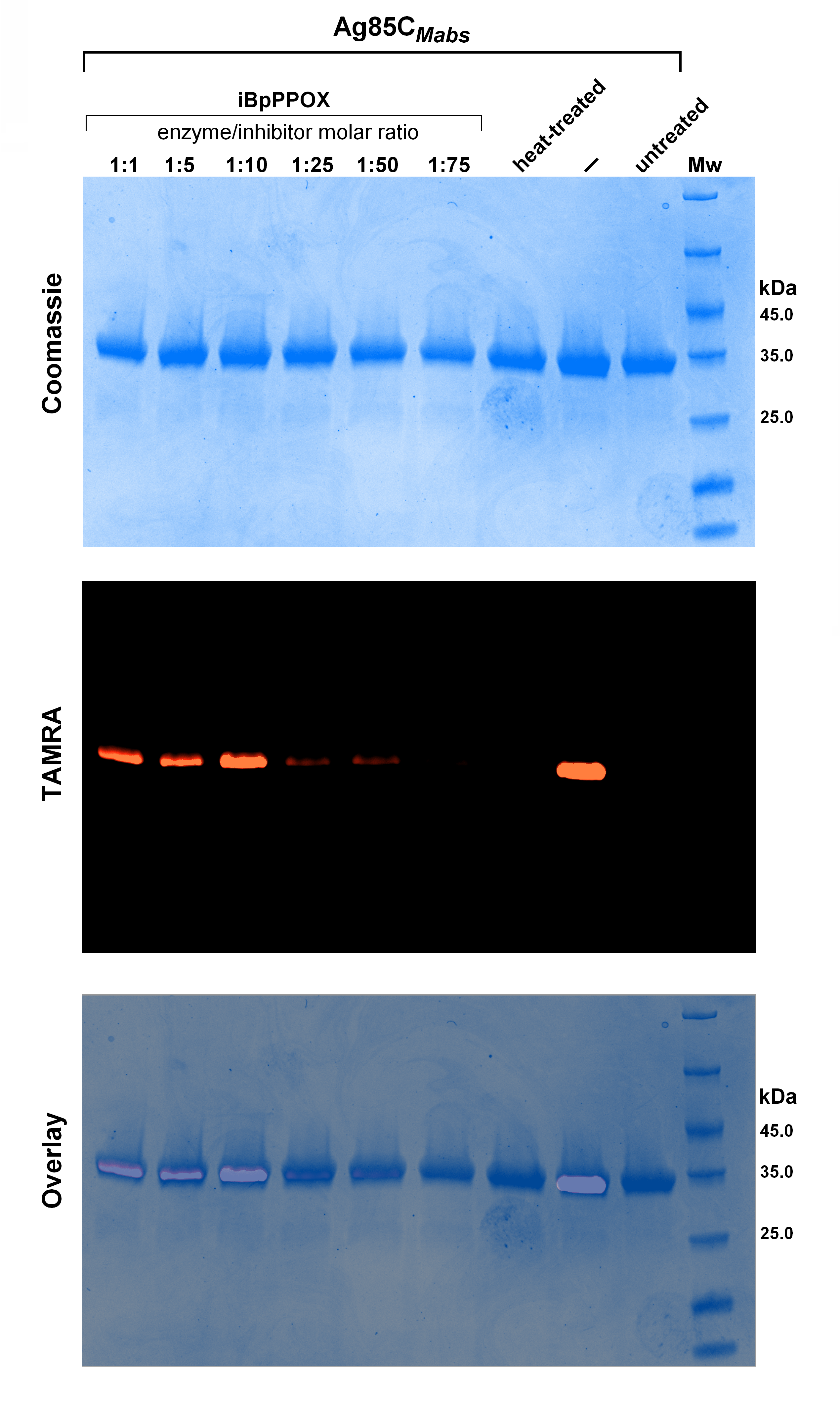

Supplement: S2 Fig — SDS-PAGE gel visualized by Coomassie blue staining (upper panel) or by in-gel fluorescence visualization (middle panel). Superimposition of both images is reported in the lower panel. Molecular weights were derived from the Unstained Protein Molecular Weight Marker (Euromedex). (TIF) [file pone.0238178.s003.tif]
